# Effect of dietary inflammatory potential on the aging acceleration for cardiometabolic disease: A population-based study

**DOI:** 10.3389/fnut.2022.1048448

**Published:** 2022-12-02

**Authors:** Yuanlong Hu, Xiaojie Wang, Jiaming Huan, Lei Zhang, Lin Lin, Yuan Li, Yunlun Li

**Affiliations:** ^1^First Clinical Medical College, Shandong University of Traditional Chinese Medicine, Jinan, Shandong, China; ^2^Shandong Province Engineering Laboratory of Traditional Chinese Medicine Precise Diagnosis and Treatment of Cardiovascular Disease, Shandong University of Traditional Chinese Medicine, Jinan, Shandong, China; ^3^Faculty of Chinese Medicine, Macau University of Science and Technology, Taipa, Macau, China; ^4^College of Traditional Chinese Medicine, Shandong University of Traditional Chinese Medicine, Jinan, China; ^5^Innovative Institute of Chinese Medicine and Pharmacy, Shandong University of Traditional Chinese Medicine, Jinan, China; ^6^Experimental Center, Shandong University of Traditional Chinese Medicine, Jinan, Shandong, China; ^7^Key Laboratory of Traditional Chinese Medicine Classical Theory, Ministry of Education, Shandong University of Traditional Chinese Medicine, Jinan, Shandong, China; ^8^Shandong Provincial Key Laboratory of Traditional Chinese Medicine for Basic Research, Shandong University of Traditional Chinese Medicine, Jinan, Shandong, China; ^9^Department of Cardiovascular, Affiliated Hospital of Shandong University of Traditional Chinese Medicine, Jinan, Shandong, China

**Keywords:** dietary inflammatory potential, dietary inflammatory index, aging acceleration, biological aging, cardiometabolic disease

## Abstract

**Background/Aim:**

Optimized dietary patterns have been considered an important determinant of delaying aging in cardiometabolic disease (CMD). Dietary pattern with high-level dietary inflammatory potential is a key risk factor for cardiometabolic disease, and has drawn increasing attention. The aim of this study was to investigate whether dietary pattern with high dietary inflammatory potential was associated with aging acceleration in cardiometabolic disease.

**Materials and methods:**

We analyzed the cross-sectional data from six survey cycles (1999–2000, 2001–2002, 2003–2004, 2005–2006, 2007–2008, and 2009–2010) of the National Health and Nutritional Examination Surveys (NHANES). A total of 16,681 non-institutionalized adults and non-pregnant females with CMD were included in this study. Dietary inflammatory index (DII) was used to assess the dietary inflammatory potential. The two age acceleration biomarkers were calculated by the residuals from regressing chronologic age on Klemera-Doubal method biological age (KDM BioAge) or Phenotypic Age (PhenoAge), termed “KDMAccel” and “PhenoAgeAccel.” A multivariable linear regression accounting for multistage survey design and sampling weights was used in different models to investigate the association between DII and aging acceleration. Four sensitivity analyses were used to ensure the robustness of our results. Besides, we also analyzed the anti-aging effects of DASH-type dietary pattern and “Life’s Simple 7”.

**Results:**

For 16,681 participants with CMD, compared with the first tertile of DII after adjusting for all potential confounders, the patients with second tertile of DII showed a 1.02-years increase in KDMAccel and 0.63-years increase in PhenoAgeAccel (KDMAccel, β = 1.02, 95% CI = 0.64 to 1.41, *P* < 0.001; PhenoAgeAccel, β = 0.63, 95% CI = 0.44 to 0.82, *P* < 0.001), while the patients with the third tertile of DII showed a 1.48-years increase in KDMAccel and 1.22-years increase in PhenoAgeAccel (KDMAccel, β = 1.48, 95% CI = 1.02 to 1.94, *P* < 0.001; PhenoAgeAccel, β = 1.22, 95% CI = 1.01 to 1.43, *P* < 0.001). In addition, DASH-type dietary pattern was associated with a 0.57-years reduction in KDMAccel (β = −0.57, 95% CI = −1.08 to −0.06, *P* = 0.031) and a 0.54-years reduction in PhenoAgeAccel (β = −0.54, 95% CI = −0.80 to −0.28, *P* < 0.001). The each one-unit increase in CVH score was associated with a 1.58-years decrease in KDMAccel (β = −1.58, 95% CI = −1.68 to −1.49, *P* < 0.001) and a 0.36-years in PhenoAgeAccel (β = −0.36, 95% CI = −0.41 to −0.31, *P* < 0.001).

**Conclusion:**

Among CMD, the dietary pattern with high dietary inflammatory potential was association with aging acceleration, and the anti-aging potential of DASH-type dietary pattern and “Life’s Simple 7” should also be given attention, but these observations require future prospective validation.

## Introduction

The spectrum of cardiometabolic disease (CMD) initiates with insulin resistance, and eventually progresses to cardiovascular diseases (CVD) or diabetes mellitus (DM), following metabolic syndrome (MetS) and prediabetes (1). The cardiometabolic disease represents a major disease burden globally, and it is the leading cause of death and disability worldwide (2–4). Accelerated aging was considered as a key driver of pathological progression for cardiometabolic disease (5–8). Epidemiological evidence suggested that age acceleration defined by the epigenetic clock was positively associated with increased cardiometabolic risk factors and cardiovascular disease risk (9–11). Several additional studies based on shortened leukocyte telomere length also confirmed that age acceleration was causally associated with coronary heart disease susceptibility (12), coronary artery calcification risk (13), and glycemic progression in type 2 DM (14). Several advancements toward anti-aging therapies have been made in the maintenance of cardiometabolic health (15, 16).

Inflammation serves as a core pillar of aging (17). Aging is accompanied by the development of chronic low-grade systemic inflammation, termed “inflammaging” (18) or “senoinflammation” (19), and the decrease of the adaptive immune response, termed “immunosenescence.” In the context of immunosenescence, the reinforced stimulation of the innate immune response leads to expansion of the senescence-associated secretory phenotype (SASP), which was the main source of “inflammaging” or “senoinflammation” (20, 21). Substantial evidence suggests chronic low-grade systemic inflammation is an independent risk factor for age-related cardiometabolic disease (22–24). Thus, control of inflammation is considered a key intervention for anti-aging and treatment of cardiometabolic disease (25).

Due to degeneracy and evolutionary conservation between immune and metabolic pathways, dietary patterns with nutrient excess and processing defects can trigger inflammatory responses (18, 26–28). The excess intakes of energy, carbohydrate, and dietary fat were considered as the main source of dietary inflammatory potential. It is well-established that calorie restriction (29), low-fat diet (30), and low-carbohydrate diet (31) decreased low-grade systematic inflammation in patients with cardiometabolic disease. Previous clinical evidence suggests the significant effect of dietary patterns with high dietary inflammatory potential on an increased risk of cardiovascular disease (CVD), cardiovascular events, and related mortality (32–34). Besides, dietary inflammatory potential affects glucose and lipid metabolism negatively in both adults and children (35–37). However, it is unclear whether dietary inflammatory potential has a positive effect on aging acceleration for cardiometabolic disease.

Based on the above analysis, the following hypothesis is proposed: dietary inflammatory potential promoted accelerated aging of cardiometabolic disease. Herein, we designed an observational U.S. population-based study to verify the above hypothesis.

## Materials and methods

### Study population

The National Health and Nutrition Examination Survey (NHANES) began in the early 1960s and survey the health and nutritional status of the U.S. population through a complex, stratified, multistage probability cluster sampling design (38, 39). This study included a total of 16,681 non-institutionalized adults and non-pregnant females with cardiometabolic disease (CMD) from six NHANES survey cycles (1999–2000, 2001–2002, 2003–2004, 2005–2006, 2007–2008, and 2009–2010). NHANES was approved by the Institutional Review Board of NCHS (Protocol #98-12 and Protocol #2005-06^1^).

In this study, the cardiometabolic disease was defined as stages 2 to 4 of the Cardiometabolic Disease Staging (CMDS) system, which includes metabolic syndrome, prediabetes, diabetes mellitus, and cardiovascular disease (1). Metabolic syndrome was defined according to Adult Treatment Panel III (ATP III) (40). Cardiovascular disease was ascertained by self-reported coronary heart disease, angina, congestive heart failure, heart attack, or stroke. DM was identified by fasting glucose ≥ 126 mg/dl, 2-h oral glucose tolerance test (OGTT) glucose ≥ 200 mg/dl, self-reported DM, or currently taking anti-diabetic medication. Prediabetes was identified by fasting glucose ≥ 100 mg/dl or 2-h OGTT glucose ≥ 140 mg/dl.

### Outcome measurements

We used two methods to quantify biological aging (BioAge) measures, including the Klemera-Doubal method biological age (KDM BioAge) (41) and Phenotypic Age (PhenoAge) (42). The BioAge R package^2^ allows the user to parametrize KDM and PhenoAge algorithms using custom sets of biomarkers, then project these algorithms onto new datasets (43). We used a biomarker set provided by the developers of the BioAge R package to calculate KDM BioAge and PhenoAge, including albumin, alkaline phosphatase, c-reactive protein, total cholesterol, creatinine, HbA1c, systolic blood pressure, blood urea nitrogen, uric acid, lymphocyte percent, mean cell volume, and white blood cell count (43). The residuals from regressing chronologic age on biological age were adopted to reflect the aging acceleration, termed “KDMAccel” and “PhenoAgeAccel” (44). Detailed calculation formulas of biological aging and aging acceleration were provided in the Supplementary methods 2.1, 2.2.

### Exposure measurements

The overall effects of diet on chronic inflammation were quantified using the Dietary Inflammatory Index (DII), as previously described (45). In this study, 28 available daily nutrients data derived from the NHANES dataset were used to calculate the DII, including alcohol, β-carotene, caffeine, carbohydrate, cholesterol, energy, protein, total fat, dietary fiber, folic acid, mono-unsaturated fatty acids (MUFA), niacin, n-3 fatty acids, n-6 fatty acids, polyunsaturated fatty acid (PUFA), riboflavin, saturated fat, thiamin, vitamin A, vitamin B6, vitamin B12, vitamin C, vitamin D, vitamin E, magnesium, selenium, iron, and zinc. Detailed calculation for DII was described in the Supplementary method 2.3. DII from lowest to highest indicates the dietary inflammatory potential on a contiguous scale from maximal anti-inflammatory to maximal pro-inflammatory (45).

In addition, the “Life’s Simple Seven” and Dietary Approaches to Stop Hypertension (DASH) type dietary pattern was recommended as a dietary goal of ideal cardiovascular health by the American Heart Association (46). A DASH score was calculated according to prior work by Mellen et al. (47). Dietary pattern with a DASH score greater than or equal to 4.5 were defined as DASH type dietary pattern. To measure “Life’s Simple Seven,” the ideal cardiovascular health score (CVH score) was calculated based on the method created by Foraker, et al. (48). Higher CVH scores means better cardiovascular health level.

The collection of respondents’ food intake was conducted by 24-h dietary recall interview format, of which the computer-assisted dietary interview system (CADI) was used until 2002, and the Automated Multiple-Pass Method (AMPM) was used thereafter. It should be noted that the collection of data in the NHANES survey cycles “1999–2000” and “2001–2002” were based on single 24-h dietary recall by a structured face-to-face interview. Since 2002, a second collection was conducted by telephone interview 3 to 10 days following the first collection (38). Dietary intake was converted to nutrient intake based on the USDA’s Food and Nutrient Database for Dietary Studies (FNDDS). For two 24-h recalls, the mean daily nutrient intake was used to analyze. Extreme energy intake was considered as under- or over-reporting to be excluded, including extreme low (800 kcal/day in male and 600 kcal/day in female) and high (8,000 kcal/day in male and 6,000 kcal/day in female) value (49).

### Covariates measurements

The variables that influenced the effect of DII on biological age acceleration were considered covariates, and the variables to calculate KDM BioAge and PhenoAge were excluded. Nineteen variables were included as covariates in the final analysis, including age, sex, ethnicity, smoking status, household income, body mass index (BMI), dietary energy intake, comorbidity burden, CMDS, diabetes mellitus (DM), metabolic syndrome (MetS), hyperlipidemia, chronic kidney disease (CKD), asthma, hypertension, cardiovascular disease (CVD), anti-hypertensive drug use, and anti-hyperlipidemic drug use.

The patients with CKD were identified by the KDIGO (Kidney Disease: Improving Global Outcomes) Clinical Practice Guideline for the Management of Glomerular Diseases (50). Hypertension was identified by average systolic blood pressure (SBP) > 140 mmHg, average diastolic blood pressure (DBP) ≥ 90 mmHg, or currently taking anti-hypertensive medication. The Charlson comorbidity index (CCI) was used to assess comorbidity burden for each individual, which was the sum of the weights of each comorbidity (51, 52). Comorbidities were identified by asking “Have you ever been told that you have the illness” for the calculation of CCI, except for DM. DM was identified according to the definition in this study. Detailed comorbidity weights were provided in the Supplementary method 2.4.

### Statistical analysis

Dietary inflammatory index (DII) was analyzed as categorized variables and re-analyzed as continuous. DII was classified into three groups according to the tertile points. DASH type dietary pattern was analyzed as categorized variables, and CVH score was analyzed as continuous. All analyses were weighted by the survey R package (version 4.1-1) to account for the complex sampling design. For main analyses, participants with missing values were excluded.

A linear regression model was applied to examine the linear relationships between exposure (DII, DASH, and CVH score) and aging acceleration. The multivariable linear regression was used to adjust for potential confounders. Multivariable linear regression models were built with three levels of adjustment: model 1 adjusted for age, sex, ethnicity, and household income; model 2 adjusted for sex, dietary energy intake, and BMI; model 3 adjusted for age, sex, ethnicity, household income, smoking status, BMI, total energy intake, CCI, CVD, hypertension, DM, MetS, hyperlipidemia, asthma, CKD, anti-hypertensive drug use, and anti-hyperlipidemic drug use. Trend tests were performed by entering the median value of each tertile of DII as a continuous variable in the linear regression models. Besides, we exported the possible non-linear effect of DII on aging acceleration using a multivariable linear regression model with a restricted cubic splines (RCS) model.

We conducted four sensitivity analyses to ensure the robustness of our results. Firstly, to evaluate whether using logistic regression analyses instead of linear regression models would change overall conclusions, the “KDMAccel” and “PhenoAgeAccel” were divided into two categories: accelerated aging (KDMAccel > 0 or PhenoAgeAccel > 0) and non-accelerated aging (KDMAccel ≤ 0 or PhenoAgeAccel ≤ 0), then we re-examined the association of DII with aging acceleration. Secondly, In the absence of consideration of complex sampling designs, we reanalyzed the association between DII and aging acceleration. Thirdly, in order to potential bias from missing values, we imputed the missing value by random forest-based missForest algorithm using the missForest R package (version 1.4) (53). Fourthly, subgroup analyses were performed by age (< 60 and ≥ 60 years), sex, ethnicity, household income, BMI (< 30 and ≥ 30 kg/m^2^), smoking status, CCI (0 and > 0), CMDS, dysglycemia, hyperlipidemia, MetS, hypertension, and CVD, and the Wald test was used to test the cross-product interaction term.

All analyses were performed in R (version 4.1.2, R Core Team) using RStudio (version 2022.07.1 Build 554, RStudio, PBC, Boston, MA). A two-sided *P*-value < 0.05 was considered statistically significant in all analyses.

## Results

### Population characteristics

**FIGURE 1:**
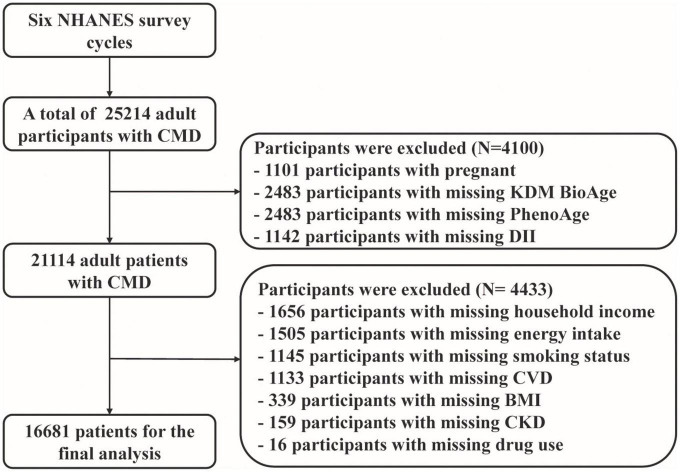
Flowchart of conforms the analytic sample.

A total of 16,681 participants with cardiovascular disease were included in the analysis (Figure 1), which represented 114.7 million non-institutionalized residents of the United States (Supplementary Table 1). Of them, 48.2% were male, and 51.8% were female. Their average age was 53.1 ± 17.7 years (Table 1). We calculated two biological age measures, the KDM BioAge and PhenoAge, both of which were significantly positively correlated with chronological age (Supplementary Table 1 and Supplementary Figure 1). Considering the complex sampling design, the average KDM and PhenoAge were 45.62 ± 18.05 and 47.07 ± 17.27, respectively (Supplementary Table 1). The mean intakes for nutrient components used to calculating DII were described in Supplementary Table 2. Only 11.8% of participants followed a DASH-type eating pattern, and the average CVH score was 7.35 ± 2.14 (Table 1).

**TABLE 1:** Characteristics of participants across the tertiles of the dietary inflammatory index.

Characteristics	Total (*N* = 16,681)	First tertile (*N* = 5,561)	Second tertile (*N* = 5,560)	Third tertile (*N* = 5,560)	*P*-value
Age, years	53.1 (17.7)	53.3 (17.1)	53.0 (17.8)	52.9 (18.1)	0.484
**Sex, %**					< 0.001***
Female	8647(51.8%)	2203(39.6%)	2919(52.5%)	3525(63.4%)	
Male	8034(48.2%)	3358(60.4%)	2641(47.5%)	2035(36.6%)	
**Ethnicity, %**					< 0.001***
White	8678(52.0%)	3080(55.4%)	2848(51.2%)	2750(49.5%)	
Black	3095(18.6%)	784(14.1%)	1046(18.8%)	1265(22.8%)	
Mexican	3371(20.2%)	1203(21.6%)	1130(20.3%)	1038(18.7%)	
Others	1537(9.21%)	494(8.88%)	536(9.64%)	507(9.12%)	
**Household income, %**					< 0.001***
≤ 130% FPL	4813(28.9%)	1291(23.2%)	1561(28.1%)	1961(35.3%)	
130–350% FPL	6585(39.5%)	2081(37.4%)	2229(40.1%)	2275(40.9%)	
>350% FPL	5283(31.7%)	2189(39.4%)	1770(31.8%)	1324(23.8%)	
**Smoking status, %**					< 0.001***
Never	8442(50.6%)	2879(51.8%)	2870(51.6%)	2693(48.4%)	
Former	4734(28.4%)	1760(31.6%)	1554(27.9%)	1420(25.5%)	
Current	3505(21.0%)	922(16.6%)	1136(20.4%)	1447(26.0%)	
BMI, kg/m^2^	29.8 (6.33)	29.3 (5.92)	30.0 (6.47)	30.1 (6.56)	< 0.001***
Energy intake, kcal/day	2007 (876)	2514 (989)	1956 (681)	1551 (627)	< 0.001***
**CMDS, %**					< 0.001***
Stage 2	9966(59.7%)	3405(61.2%)	3311(59.6%)	3250(58.5%)	
Stage 3	2164(13.0%)	783(14.1%)	728(13.1%)	653(11.7%)	
Stage 4	4551(27.3%)	1373(24.7%)	1521(27.4%)	1657(29.8%)	
CCI	1.05 (1.47)	0.99 (1.44)	1.02 (1.44)	1.13 (1.52)	< 0.001***
**Hypertension, %**					0.452
No	7945(47.6%)	2686(48.3%)	2637(47.4%)	2622(47.2%)	
Yes	8736(52.4%)	2875(51.7%)	2923(52.6%)	2938(52.8%)	
MetS, %					0.002**
No	11075(66.4%)	3787(68.1%)	3671(66.0%)	3617(65.1%)	
Yes	5606(33.6%)	1774(31.9%)	1889(34.0%)	1943(34.9%)	
**Dysglycemia, %**					< 0.001***
Normal	12404(74.4%)	4215(75.8%)	4127(74.2%)	4062(73.1%)	
IGT	301(1.80%)	100(1.80%)	83(1.49%)	118(2.12%)	
IFG	719(4.31%)	263(4.73%)	241(4.33%)	215(3.87%)	
DM	3257(19.5%)	983(17.7%)	1109(19.9%)	1165(21.0%)	
**Hyperlipidemia, %**					0.007**
No	2850(17.1%)	1015(18.3%)	944(17.0%)	891(16.0%)	
Yes	13831(82.9%)	4546(81.7%)	4616(83.0%)	4669(84.0%)	
**Asthma, %**					0.004**
No	14438(86.6%)	4870(87.6%)	4818(86.7%)	4750(85.4%)	
Yes	2243(13.4%)	691(12.4%)	742(13.3%)	810(14.6%)	
**CKD, %**					< 0.001***
No	13104(78.6%)	4482(80.6%)	4395(79.0%)	4227(76.0%)	
Yes	3577(21.4%)	1079(19.4%)	1165(21.0%)	1333(24.0%)	
**CVD, %**					< 0.001***
No	14283(85.6%)	4862(87.4%)	4770(85.8%)	4651(83.7%)	
Yes	2398(14.4%)	699(12.6%)	790(14.2%)	909(16.3%)	
**Drug use, %**					
Anti-hypertensive	2725(16.3%)	936(16.8%)	945(17.0%)	844(15.2%)	0.016*
Anti-hyperlipidemic	3391(20.3%)	1119(20.1%)	1142(20.5%)	1130(20.3%)	0.861
KDM BioAge, years	49.6 (19.7)	49.1 (18.5)	49.7 (19.8)	50.2 (20.9)	0.009**
KDMAccel, years	−3.43(10.5)	−4.24(9.85)	−3.31(10.3)	−2.73(11.2)	< 0.001***
PhenoAge, years	51.0 (18.7)	50.8 (18.0)	50.9 (18.9)	51.3 (19.2)	0.240
PhenoAgeAccel	−2.09(5.13)	−2.54(4.90)	−2.10(5.16)	−1.62(5.30)	< 0.001***
**DASH, %**					< 0.001***
No	14705(88.2%)	4506(81.0%)	5015(90.2%)	5184(93.2%)	
Yes	1976(11.8%)	1055(19.0%)	545(9.80%)	376(6.76%)	
CVH score	7.35 (2.14)	7.83 (2.09)	7.30 (2.12)	6.92 (2.12)	

CI, confidence interval; FPL, federal poverty level; IFG, Impaired Fasting Glycaemia; IGT, Impaired glucose tolerance; DM, diabetes mellitus; CCI, Charlson Comorbidity Index; CVD, cardiovascular diseases; DM, diabetes mellitus; MetS, metabolic syndrome; CKD, chronic kidney disease; KDMAccel, KDM Acceleration; PhenoAgeAccel, PhenoAge acceleration; DASH, Dietary Approaches to Stop Hypertension; CVH Score, ideal cardiovascular health score. *P*-value: *, < 0.05; **, < 0.01; ***, < 0.001. *P*-values are calculated by ANOVA or Chi-squared tests. Cut-off values of DII tertiles were 0.9 and 2.6.

### Associations of dietary inflammatory index with aging acceleration

**FIGURE 2:**
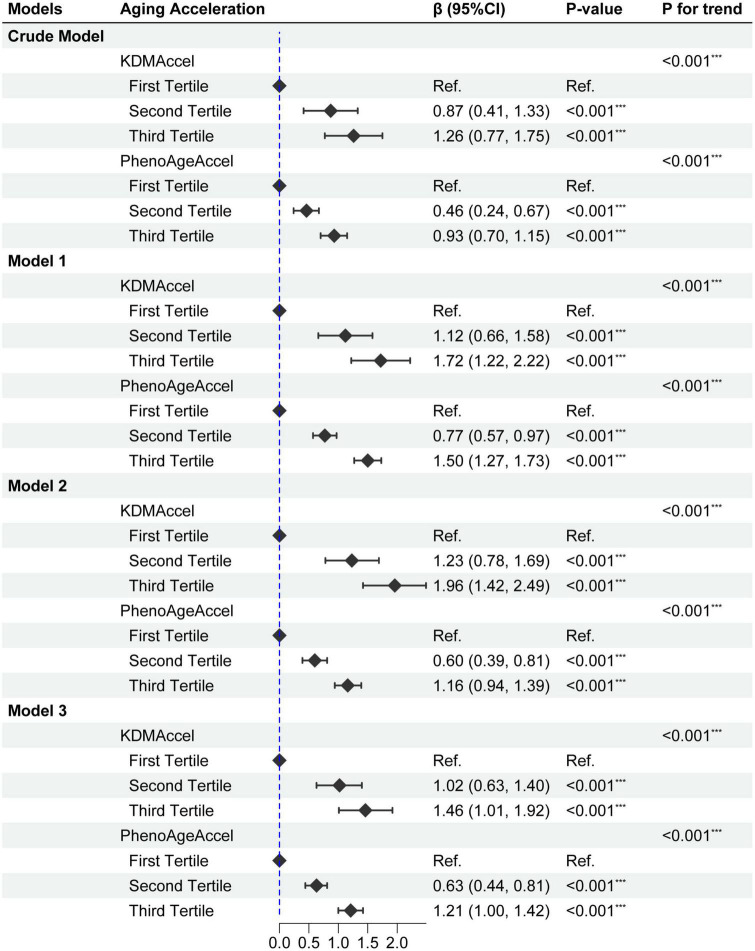
Forest plot for the effect of dietary inflammatory index (DII) on aging acceleration. Model 1 adjusted for age, sex, ethnicity, and household income; Model 2 adjusted for sex, BMI, and total energy intake; Model 3 adjusted age, sex, ethnicity, household income, smoking status, BMI, total energy intake, CCI, CVD, hypertension, dysglycemia, MetS, hyperlipidemia, asthma, CKD, anti-hypertensive drug use, and anti-hyperlipidemic drug use. *P*-value: ***, < 0.001.

Taking the complex survey design into account, univariate analysis showed that DII was significantly positively associated with KDMAccel or PhenoAgeAccel, either using the raw continuous or the categorized (tertile) measures (Figure 2 and Supplementary Table 3). Compared with the first tertile, the patients with the second tertile of DII gained 0.87 years increase in the KDMAccel (β = 0.87, 95% CI = 0.41 to 1.33, *P* < 0.001) and 0.46 years increase in the PhenoAgeAccel (β = 0.46, 95% CI = 0.24 to 0.67, *P* < 0.001), while patients with the third tertile of DII gained 1.26 years increase in the KDMAccel (β = 1.26, 95% CI = 0.77 to 1.75, *P* < 0.001) and 0.93 years increase in the PhenoAgeAccel (β = 0.93, 95% CI = 0.70 to 1.15, *P* < 0.001). For continuous DII measures, each one unit increase in DII was associated with a 0.33-years increase in KDMAccel (β = 0.33, 95% CI = 0.22 to 0.44, *P* < 0.001) and a 0.24-years increase in PhenoAgeAccel (β = 0.24, 95% CI = 0.19 to 0.29, *P* < 0.001).

**FIGURE 3:**
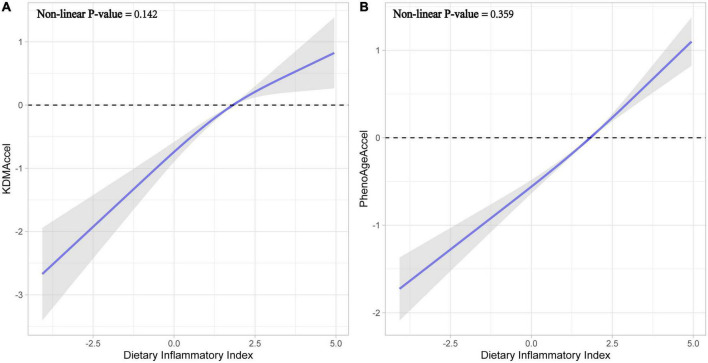
Fitting curves for restricted cubic splines models. **(A)** Effect of dietary inflammatory index on KDMAccel. **(B)** Effect of dietary inflammatory index on PhenoAgeAccel.

Subsequently, after correcting for all potential confounding variables, adjusted results were congruent with the unadjusted results either using the raw continuous or the categorized measures (Figure 2 and Supplementary Table 4). Compared with the first tertile, the patients with the second tertile of DII gained 1.02-years more KDMAccel (KDMAccel, β = 1.02, 95% CI = 0.64 to 1.41, *P* < 0.001) and 0.63-years more PhenoAgeAccel (PhenoAgeAccel, β = 0.63, 95% CI = 0.44 to 0.82, *P* < 0.001), while the patients with the third tertile of DII gained 1.48-years more KDMAccel (KDMAccel, β = 1.48, 95% CI = 1.02 to 1.94, *P* < 0.001) and 1.22-years more PhenoAgeAccel (β = 1.22, 95% CI = 1.01 to 1.43, *P* < 0.001). For continuous DII measures, each one unit increase in DII was associated with a 0.38-years increase in KDMAccel (β = 0.38, 95% CI = 0.29 to 0.48, *P* < 0.001) and a 0.32-years increase in PhenoAgeAccel (β = 0.32, 95% CI = 0.27 to 0.36, *P* < 0.001). Besides, the RCS model did not suggest any non-linear dose-response associations between DII and two biomarkers of aging acceleration (Figure 3).

Additionally, we also analyzed the effect of DASH-type dietary pattern and CVH score on KDMAccel and PhenoAgeAccel. DASH-type dietary pattern was associated with a 0.57-years reduction in KDMAccel (β = −0.57, 95% CI = −1.08 to −0.06, *P* = 0.031) and a 0.54-years reduction in PhenoAgeAccel (β = −0.54, 95% CI = −0.80 to −0.28, *P* < 0.001) after adjusting for all potential confounders (Supplementary Figure 2). The each one-unit increase in CVH score was associated with a 1.58-years decrease in KDMAccel (β = −1.58, 95% CI = −1.68 to −1.49, *P* < 0.001) and a 0.36-years in PhenoAgeAccel (β = −0.36, 95% CI = −0.41 to −0.31, *P* < 0.001) after adjusting for all potential confounders (Supplementary Figure 3).

### Sensitivity analyses

We used four different ways to perform sensitivity analyses to determine the robustness of the results. The results of subgroup analyses showed that the direction of the accelerated aging effect was consistent with main analyses across subgroups (Supplementary Tables 5–7). It is important to note that the hypertension and CVD showed an interaction with DII on the KDMAccel (hypertension, *P* for interaction < 0.001; CVD, *P* for interaction < 0.001) and PhenoAgeAccel (hypertension, *P* for interaction = 0.002; CVD, *P* for interaction < 0.001). The associations were stronger in individuals with CVD or hypertension (Supplementary Tables 5–7).

The association of DII with aging acceleration was reanalyzed in each of the following three cases: data without taking sampling weight into account (Supplementary Table 8), imputed data using the missForest methods (Supplementary Table 9), and the KDMAccel and PhenoAgeAccel redefined as categorical data based on whether it is more than 0 (Supplementary Table 10). The results of all cases showed that DII was significantly positively associated with the aging acceleration, which was similar to the main analysis.

## Discussion

This analysis in a nationally representative U.S. adult population with cardiometabolic disease revealed three main findings. First, we determined that the dietary pattern with higher DII was associated with faster biological aging. Second, the “Life’s Simple Seven” and DASH-type dietary pattern demonstrated a significant slowing effect on the speed of aging. Third, the low-DII dietary pattern was more beneficial for patients with cardiovascular disease or hypertension. The above result was simultaneously validated in the analysis with KDMAccel and PhenoAgeAccel as outcome variables. In addition, we continued with three sensitivity analyses, and the results of this analysis were in agreement with the results of the main analysis, which strengthened our confidence in the above results.

For this study, the primary challenge facing to us is how to measure the accelerated aging effect. The most common clinical outcomes in current anti-aging clinical studies mainly include age-related diseases and aging-related biomarkers (54, 55). Distinguishing from chronological age, biological age as a biomarker of aging is a promising surrogate endpoint for anti-aging therapies in clinical research, such as KDM BioAge and PhenoAge (56, 57). In two studies based on NHANES data, KDM BioAge and PhenoAge were confirmed to be robust predictors of mortality (58–60). Thus, KDM BioAge and PhenoAge are suited for measuring the accelerated aging effect in cardiometabolic disease. Although KDM BioAge and PhenoAge are significantly correlated, the recent genome-wide association study linked KDM BioAge and PhenoAge to different aspects of the aging process (61), which tend to reflect cardiometabolic risk and inflammaging, respectively. High-DII dietary pattern was a risk factor for cardiovascular metabolic risk and systemic inflammation. Therefore, KDM BioAge and PhenoAge are suitable tools for measuring aging acceleration in patients with cardiometabolic disease.

Our study based on the NHANES dataset found that a dietary pattern with higher dietary inflammatory potential was associated with a faster aging acceleration in patients with cardiometabolic disease. In addition to KDM BioAge and PhenoAge, other aging-related biomarkers also provided evidence for the effect of high DII on accelerated aging, such as telomere shortening and frailty. Telomere shortening is a biomarker of cardiovascular aging, which is the cause of replicative cellular senescence and the risk factor of cardiovascular disease (62). The evidence from the PREDIMED-NAVARRA (PREvención con DIeta MEDiterránea-NAVARRA) trial also suggested that a high-level dietary inflammatory index promoted telomere shortening in 520 participants with high cardiovascular disease risk (63). However, previous research suggested that telomere length was not significantly related to KDM BioAge (56, 64). Frailty is also an aging-related biomarker (65), which represents poor prognosis in cardiovascular disease (66). Three clinical studies suggested that a high-DII dietary pattern was associated with an increased risk of frailty (67–69). Taking all findings of the above studies on different aging markers together, high levels of dietary inflammatory potential can contribute to the acceleration of the aging process.

In the subgroup analysis, patients with cardiovascular disease or hypertension appeared to be more sensitive to the pro-aging accelerating effects of high-DII dietary patterns. We speculated that the reason was accelerated aging as the critical contributing factor leading to CVD and hypertension (60, 70). It was suggested that the low-DII dietary pattern provided a novel and promising dietary nutritional strategy for the treatment of CVD and hypertension.

Our study suggested that DASH-type dietary pattern and “Life’s Simple 7” could also have beneficial effects on aging in patients with cardiometabolic disease. The “Life’s Simple 7” and DASH type dietary pattern were defined by the American Heart Association as the ideal lifestyle and clinical factors. Two previous observational studies suggested that the individuals who follow “Life’s Simple 7” exhibited slower aging speeds among populations free of CVD from the Women’s Health Initiative (WHI) and Taiwan Biobank (TWB) (71, 72). In addition, the findings of the Framingham Heart Study Offspring Cohort showed that the DASH-type dietary pattern was associated with a lower acceleration of epigenetic age (73). The DASH-type dietary pattern has an anti-inflammatory effect (74, 75). Our findings were also in agreement with previous studies, in particular, our study provided results in the cardiometabolic disease population, stressing the importance of a DASH-type dietary pattern for aging.

Our findings have clinical implications for cardiometabolic disease. Anti-aging therapies have emerged as a new hope for the treatment of cardiometabolic disease. Optimized dietary pattern plays a major role in affecting aging and cardiometabolic health, which has long been recognized as the most potent, feasible, and safest intervention to delay aging (76). In this context, a new question urgently needs to be addressed: what dietary patterns or lifestyles can provide the benefits of slowing aging for patients with cardiometabolic diseases? Calorie restriction is a typical example of a dietary pattern with anti-aging effects (76), which has been shown to reduce cardiometabolic risk by clinical evidence from substantial randomized controlled studies (77, 78). Our study suggested that dietary inflammatory potential is a key concern and consideration in developing dietary patterns for patients with cardiometabolic disease, especially among CVD and hypertension patients. And, the anti-aging potential of DASH-type dietary pattern and “Life’s Simple 7” should also be given attention in the treatment and prevention of cardiometabolic disease.

A lot of limitations should be noted in our study. Firstly, missing data could lend to selection bias. We performed a sensitivity analysis for reanalyzing the imputed data, which supports the robustness of our study. Secondly, the inclusion of only the U.S. population in our study limits the generalization of our findings to other populations. Thirdly, our study was based on a cross-sectional NHANES dataset, which limited causal inferences. We hope that future controlled randomized controlled studies, prospective cohort studies, or mendelian randomization studies are needed to confirm our results. Fourth, the history of disease and medication were collected by self-reporting in the NHANES, which might bring selection bias to our results. Besides, the survey for dietary intake based on 24-h recall was subject to recall bias. Fifth, the measurement of aging acceleration remains somewhat different for different biological age markers, so we applied two different biological age markers and the results of both readily corroborate each other, which increases the robustness of our findings.

In summary, our study demonstrates that the dietary pattern with a higher DII was associated with faster aging acceleration in patients with cardiometabolic disease, which should receive more attention. Besides, the DASH-type dietary pattern and “Life’s Simple Seven” showed an anti-aging effect. The low-DII dietary pattern, DASH type dietary pattern, and “Life’s Simple Seven” could be recommended as potential strategies to slow the pace of aging in cardiometabolic disease populations.

## Data availability statement

Publicly available datasets were analyzed in this study. This data can be found here: https://www.cdc.gov/nchs/nhanes/index.htm.

## Author contributions

YH conducted analyses and wrote the manuscript. XW, JH, LZ, LL, and YaL collected and assembled the data. YH and YlL conceived of the study design. All authors have contributed to the interpretation of the results, have critically revised the content of the manuscript, and agreed to be accountable for all aspects of the work.

## Footnotes

1
https://www.cdc.gov/nchs/nhanes/irba98.htm

2
https://github.com/dayoonkwon/BioAge
