# Supplementary material for: Effect of dietary inflammatory potential on the aging acceleration for cardiometabolic disease: A population-based study
Source: Front Nutr. 2022 Dec 2;9:1048448. doi: 10.3389/fnut.2022.1048448 (PMC9755741; doi:10.3389/fnut.2022.1048448)
Supplement: Supplementary file 1 [file Data_Sheet_1.docx]

Supplementary Material

# Supplementary Figures and Tables

## Supplementary Figures


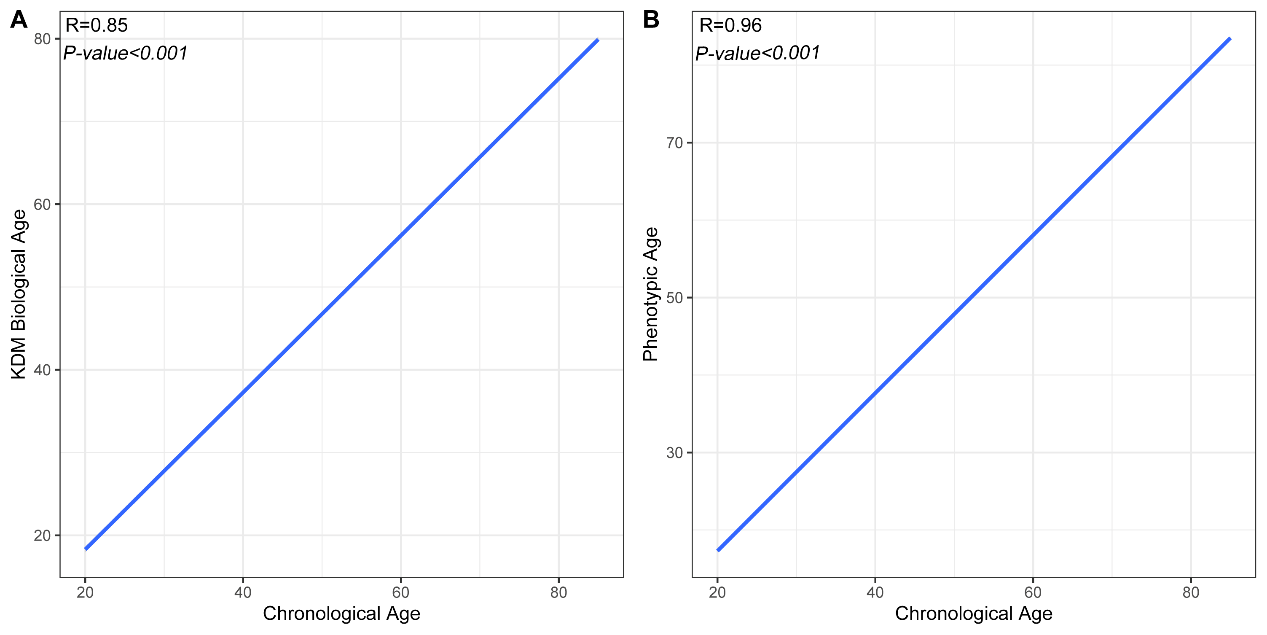


**Supplementary Figure 1.** The Pearson correlations between biological age and chronological age. (A) The Pearson correlations between KDM BioAge and chronological age. (B) The Pearson correlations between PhenoAge and chronological age.

**
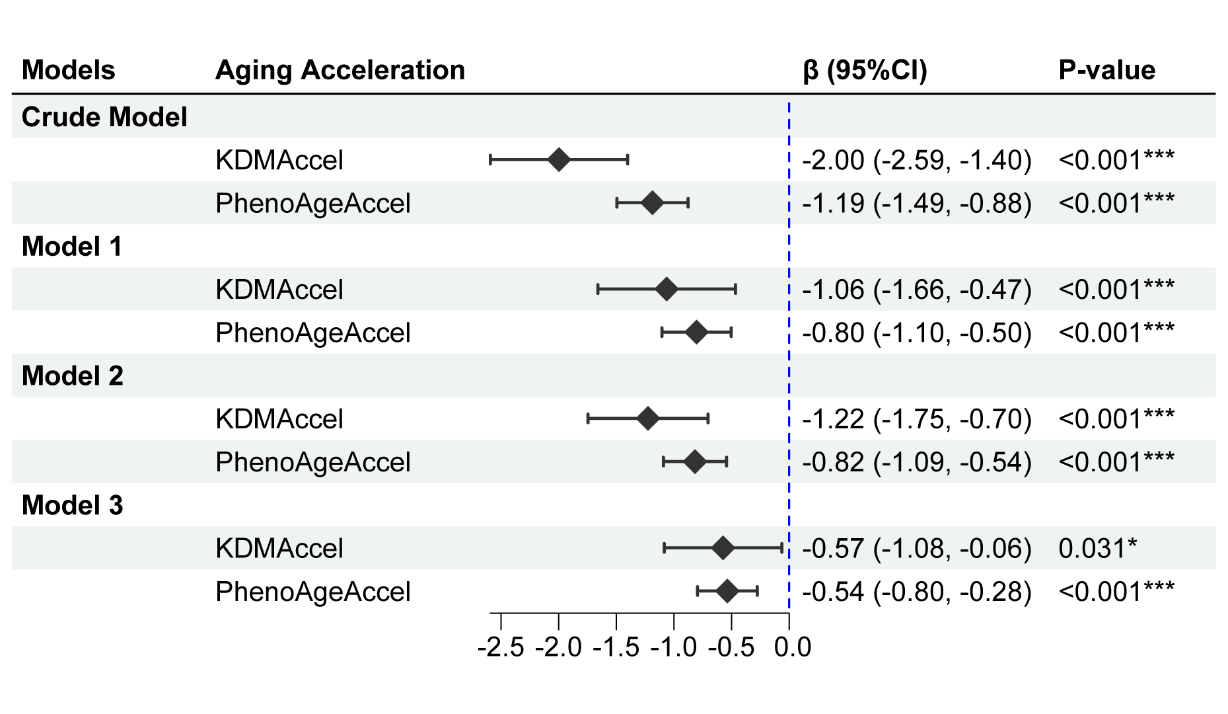
**

**Supplementary Figure 2.** The effect of DASH on KDMAccel and PhenoAgeAccel.

**
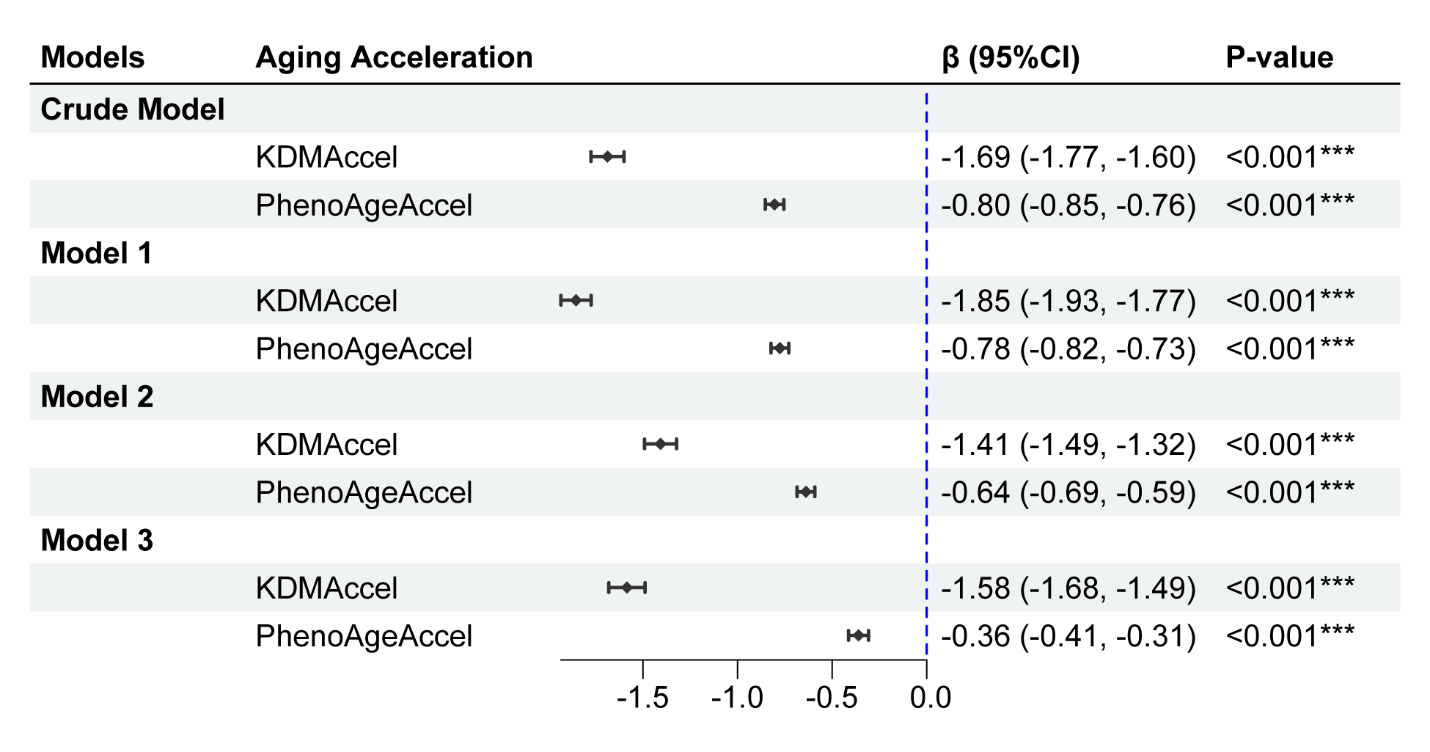
**

**Supplementary Figure 3.** The effect of Cardiovascular Health Score on KDMAccel and PhenoAgeAccel.

## Supplementary Table

**Supplementary Table 1.** Weighted characteristics of participants across the tertiles of the Dietary Inflammatory Index.

| **Characteristic** | **Total**  **(N=114650046.1)** | **First Tertile**  **(N=40254870.31)** | **Second Tertile**  **(N=38296661.9)** | **Third Tertile**  **(N=36096817.45)** | ***P*-value** |
| --- | --- | --- | --- | --- | --- |
| Age | 49.44 (16.47) | 49.90 (15.83) | 49.45 (16.61) | 48.92 (16.99) | 0.027^*^ |
| Gender, % |  |  |  |  | <0.001^***^ |
| Female | 60538769.1 (52.8) | 16161205.3 (40.1) | 20508315.5 (53.6) | 23869248.3 (66.1) |  |
| Male | 54111277.0 (47.2) | 24093665.0 (59.9) | 17788346.4 (46.4) | 12227569.2 (33.9) |  |
| Ethnicity, % |  |  |  |  | <0.001^***^ |
| White | 84327207.4 (73.6) | 30796163.2 (76.5) | 27708092.7 (72.4) | 25821255.2 (71.5) |  |
| Black | 11525957.6 (10.1) | 2874479.2 (7.1) | 3941695.6 (10.3) | 4709782.7 (13.0) |  |
| Mexican | 8345635.4 (7.3) | 3114790.2 (7.7) | 2875600.4 (7.5) | 2355244.8 (6.5) |  |
| Others | 10451245.7 (9.1) | 3469437.7 (8.6) | 3771273.3 (9.8) | 3210534.7 (8.9) |  |
| Household income, % |  |  |  |  | <0.001^***^ |
| ≤130% FBL | 23188666.6 (20.2) | 6257647.0 (15.5) | 7609054.5 (19.9) | 9320268.7 (25.8) |  |
| ≤350% FBL | 42633016.6 (37.2) | 13640858.8 (33.9) | 14383147.9 (37.6) | 14609009.9 (40.5) |  |
| >350% FBL | 48828362.9 (42.6) | 20356364.5 (50.6) | 16304459.5 (42.6) | 12167538.9 (33.7) |  |
| Smoking status, % |  |  |  |  | <0.001^***^ |
| Never | 57782730.5 (50.4) | 20925948.9 (52.0) | 19821568.5 (51.8) | 17035213.1 (47.2) |  |
| Former | 31033395.0 (27.1) | 12649914.0 (31.4) | 9796280.3 (25.6) | 8585504.3 (23.8) |  |
| Current | 25833920.6 (22.5) | 6679007.3 (16.6) | 8678813.1 (22.7) | 10476100.1 (29.0) |  |
| BMI, kg/m^2^ | 29.72 (6.37) | 29.31 (6.10) | 29.84 (6.41) | 30.06 (6.59) | <0.001^***^ |
| Total energy intake, kcal/day | 2113.19 (904.10) | 2605.05 (1013.51) | 2051.97 (703.36) | 1629.62 (646.87) | <0.001^***^ |
| CCI | 0.90 (1.36) | 0.84 (1.32) | 0.88 (1.34) | 0.97 (1.42) | <0.001^***^ |
| Dysglycemia, % |  |  |  |  | 0.177 |
| Normal | 91547597.2 (79.8) | 32355423.3 (80.4) | 30577883.0 (79.8) | 28612594.5 (79.3) |  |
| IGT | 1960838.2 (1.7) | 673113.0 (1.7) | 557019.6 (1.5) | 730705.7 (2.0) |  |
| IFG | 4510684.6 (3.9) | 1707353.1 (4.2) | 1489537.2 (3.9) | 1313794.3 (3.6) |  |
| DM | 16630926.1 (14.5) | 5518980.9 (13.7) | 5672222.1 (14.8) | 5439723.0 (15.1) |  |
| Hyperlipidemia (%) |  |  |  |  | 0.104 |
| No | 19041739.9 (16.6) | 7057625.3 (17.5) | 6323755.0 (16.5) | 5660359.6 (15.7) |  |
| Yes | 95608306.2 (83.4) | 33197245.1 (82.5) | 31972906.9 (83.5) | 30436457.8 (84.3) |  |
| CKD (%) |  |  |  |  | <0.001^***^ |
| No | 95938828.5 (83.7) | 34582034.4 (85.9) | 32025704.0 (83.6) | 29329393.6 (81.3) |  |
| Yes | 18711217.6 (16.3) | 5672835.9 (14.1) | 6270957.9 (16.4) | 6767423.8 (18.7) |  |
| Asthma (%) |  |  |  |  | 0.002^**^ |
| No | 98248760.3 (85.7) | 34761894.5 (86.4) | 33206749.2 (86.7) | 30280116.5 (83.9) |  |
| Yes | 16401285.8 (14.3) | 5492975.8 (13.6) | 5089912.7 (13.3) | 5816700.9 (16.1) |  |
| MetS_ATP (%) |  |  |  |  | 0.006^**^ |
| No | 77271532.5 (67.4) | 27957458.2 (69.5) | 25613217.6 (66.9) | 23699160.4 (65.7) |  |
| Yes | 37378513.5 (32.6) | 12297412.2 (30.5) | 12683444.3 (33.1) | 12397657.1 (34.3) |  |
| CMDS |  |  |  |  | 0.053 |
| Stage 2 | 75062003.9 (65.5) | 26695006.8 (66.3) | 25006650.8 (65.3) | 23358649.9 (64.7) |  |
| Stage 3 | 15256502.7 (13.3) | 5610282.3 (13.9) | 5090952.7 (13.3) | 4555267.7 (12.6) |  |
| Stage 4 | 24331539.5 (21.2) | 7949581.2 (19.7) | 8199058.4 (21.4) | 8182899.8 (22.7) |  |
| Hypertension (%) |  |  |  |  | 0.690 |
| No | 61075663.0 (53.3) | 21250660.6 (52.8) | 20594441.4 (53.8) | 19230561.1 (53.3) |  |
| Yes | 53574383.1 (46.7) | 19004209.7 (47.2) | 17702220.5 (46.2) | 16866256.4 (46.7) |  |
| CVD |  |  |  |  | <0.001^***^ |
| No | 101603362.1 (88.6) | 36228025.3 (90.0) | 33945504.9 (88.6) | 31428135.5 (87.1) |  |
| Yes | 13046684.0 (11.4) | 4026845.0 (10.0) | 4351157.0 (11.4) | 4668681.9 (12.9) |  |
| Anti-Hypertensive, % |  |  |  |  | 0.010^*^ |
| No | 98023651.2 (85.5) | 34087109.2 (84.7) | 32545920.0 (85.0) | 31388925.7 (87.0) |  |
| Yes | 16626394.8 (14.5) | 6167761.1 (15.3) | 5750741.9 (15.0) | 4707891.8 (13.0) |  |
| Anti-Diabetic, % |  |  |  |  | 0.156 |
| No | 104998286.3 (91.6) | 37145791.2 (92.3) | 34878323.8 (91.1) | 32972474.8 (91.3) |  |
| Yes | 9651759.8 (8.4) | 3109079.1 (7.7) | 3418338.1 (8.9) | 3124342.6 (8.7) |  |
| Anti-Hyperlipidemic, % |  |  |  |  | 0.771 |
| No | 93590530.0 (81.6) | 32741638.9 (81.3) | 31241205.2 (81.6) | 29605989.4 (82.0) |  |
| Yes | 21059516.1 (18.4) | 7513231.4 (18.7) | 7055456.7 (18.4) | 6490828.0 (18.0) |  |
| KDM BioAge | 45.62 (18.05) | 45.39 (16.61) | 45.81 (18.34) | 45.66 (19.24) | 0.612 |
| KDMAccel | -3.83 (9.57) | -4.52 (8.94) | -3.65 (9.51) | -3.25 (10.23) | <0.001^***^ |
| PhenoAge | 47.07 (17.27) | 47.09 (16.36) | 47.09 (17.58) | 47.03 (17.92) | 0.982 |
| PhenoAgeAccel | -2.37 (4.81) | -2.82 (4.57) | -2.36 (4.90) | -1.89 (4.94) | <0.001^***^ |
| DASH |  |  |  |  | <0.001^***^ |
| No | 102914107.2 (89.8) | 33416231.4 (83.0) | 35373322.8 (92.4) | 34124553.0 (94.5) |  |
| Yes | 11735938.9 (10.2) | 6840335.3 (17.0) | 2923339.1 (7.6) | 1972264.4 (5.5) |  |
| CVH Score | 7.59 (2.13) | 8.05 (2.07) | 7.54 (2.11) | 7.14 (2.12) | <0.001^***^ |

**Abbreviations:** CI = confidence interval; FPL = federal poverty level; CHF = congestive heart failure; IFG = Impaired Fasting Glycaemia; IGT = Impaired Glucose Tolerance; DM = diabetes mellitus; KDMAccel = KDM Acceleration; PhenoAgeAccel = PhenoAge acceleration; CVH Score = ideal cardiovascular health score.

**Notes:** *, <0.05; **, <0.01; ***, <0.001. P-values are calculated by ANOVA or Chi-squared-tests. Cut-off values of DII tertiles were 0.9 and 2.6.

**Supplementary Table 2.** Unweighted and Weighted characteristics of participants for DII components.

| **Components** | **Unweighted value** | **Weighted value** |
| --- | --- | --- |
| **Absolute Intake** |  |  |
| Total energy, kcal/day | 2007.2 (876.12) | 2113.19 (904.10) |
| Protein, g/day | 77.92 (36.65) | 81.44 (37.73) |
| Carbohydrate, g | 247.89 (113.75) | 257.22 (117.45) |
| Dietary fiber, g/day | 15.87 (9.08) | 15.93(9.00) |
| Total fat, g/day | 75.14 (40.02) | 80.46(41.86) |
| Saturated fat, g/day | 24.48 (14.31) | 26.46(15.16) |
| MUFA, g/day | 27.81 (15.75) | 29.75(16.46) |
| PUFA, g/day | 16.07 (9.74) | 17.05(10.17) |
| Cholesterol, mg/day | 283.34 (207.48) | 287.14(208.42) |
| n-3 fatty acids, g/day | 0.14 (0.34) | 0.14 (0.35) |
| Eicosapentaenoic (20:5), g/day | 0.04 (0.12) | 0.04 (0.13) |
| Docosapentaenoic (22:5), g/day | 0.02 (0.04) | 0.02 (0.04) |
| Docosahexaenoic (22:6), g/day | 0.08 (0.19) | 0.08 (0.20) |
| n-6 fatty acids, g/day | 15.74 (9.59) | 16.70 (10.03) |
| Octadecadienoic (18:2), g/day | 14.17 (8.76) | 15.05 (9.15) |
| Octadecatrienoic (18:3), g/day | 1.41 (0.93) | 1.50 (0.98) |
| Octadecatetraenoic (18:4), g/day | 0.01 (0.03) | 0.01 (0.03) |
| Eicosatetraenoic (20:4), g/day | 0.14 (0.12) | 0.14 (0.12) |
| Vitamin A, mcg/day | 610.27 (680.38) | 627.71 (619.31) |
| Beta-carotene, mcg/day | 2072.74 (3038.54) | 2094.75 (2994.16) |
| Vitamin B1, mg/day | 1.56 (0.81) | 1.63 (0.85) |
| Vitamin B2, mg/day | 2.04 (1.10) | 2.16 (1.12) |
| Niacin, mg/day | 22.94 (11.94) | 24.05 (12.26) |
| Vitamin B6, mg/day | 1.87 (1.09) | 1.93 (1.11) |
| Folic acid, mcg/day | 182.36 (156.95) | 190.13 (161.35) |
| Vitamin B12, mcg/day | 5.18 (7.71) | 5.35 (7.18) |
| Vitamin C, mg/day | 90.51 (89.15) | 87.35 (84.35) |
| Vitamin D, mcg/day | 4.63 (4.20) | 4.78 (4.44) |
| Vitamin E, mg/day | 98.25 (421.1) | 104.66 (404.80) |
| Magnesium, mg/day | 274.35 (127.89) | 285.22 (131.18) |
| Iron, mg/day | 14.94 (8.16) | 15.47 (8.34) |
| Zinc, mg/day | 11.41 (8.25) | 12.02 (8.38) |
| Selenium, mcg/day | 104.75 (55.44) | 108.91 (56.25) |
| Caffeine, mg/day | 161.77 (203.84) | 192.62 (229.30) |
| Alcohol, g/day | 8.03 (25.19) | 9.22 (27.31) |
| **Percent Energy** |  |  |
| Carbohydrate, % total energy/day | 50.03 (10.33) | 49.28 (10.39) |
| Protein, % total energy/day | 15.86 (4.38) | 15.74 (4.39) |
| Total fat, % total energy/day | 33.23 (8.17) | 33.85 (8.25) |
| Alcohol, % total energy/day | 2.36 (5.96) | 2.63 (6.17) |

**Notes:** The n-3 fatty acids intake was the sum of Eicosapentaenoic (20:5), Docosapentaenoic (22:5), and Docosahexaenoic (22:6). The n-6 fatty acids intake was the sum of Octadecadienoic (18:2), Octadecatrienoic (18:3), Octadecatetraenoic (18:4), and Eicosatetraenoic (20:4).

**Supplementary Table 3. Univariate analyses for the effect on aging acceleration.**

| **Characteristic** | **KDMAccel** | | **PhenoAgeAccel** | |
| --- | --- | --- | --- | --- |
|  | **β (95% CI)** | ***P*-value** | **β (95% CI)** | ***P*-value** |
| DII- Tertile |  |  |  |  |
| First Tertile | Ref. | Ref. | Ref. | Ref. |
| Second Tertile | 0.87 (0.41, 1.33) | <0.001^***^ | 0.46 (0.24, 0.67) | <0.001^***^ |
| Third Tertile | 1.26 (0.77, 1.75) | <0.001^***^ | 0.93 (0.70, 1.15) | <0.001^***^ |
| DII | 0.33 (0.22, 0.44) | <0.001^***^ | 0.24 (0.19, 0.29) | <0.001^***^ |
| Age, years | -0.07 (-0.08, -0.06) | <0.001^***^ | 0.01 (0, 0.01) | 0.013^*^ |
| Gender |  |  |  |  |
| Female | Ref. | Ref. | Ref. | Ref. |
| Male | 3.18 (2.86, 3.5) | <0.001^***^ | 2.65 (2.48, 2.81) | <0.001^***^ |
| Ethnicity |  |  |  |  |
| White | Ref. | Ref. | Ref. | Ref. |
| Black | 3.67 (3.23, 4.11) | <0.001^***^ | 1.09 (0.81, 1.36) | <0.001^***^ |
| Mexican | 1.09 (0.67, 1.51) | <0.001^***^ | -0.46 (-0.78, -0.13) | 0.007^**^ |
| Others | 0.56 (-0.1, 1.21) | 0.100 | -0.44 (-0.82, -0.07) | 0.023^*^ |
| Household income |  |  |  |  |
| ≤130% FPL | Ref. | Ref. | Ref. | Ref. |
| ≤350% FPL | -1.28 (-1.78, -0.79) | <0.001^***^ | -0.46 (-0.70, -0.21) | <0.001^***^ |
| >350% FPL | -2.13 (-2.62, -1.65) | <0.001^***^ | -0.98 (-1.27, -0.69) | <0.001^***^ |
| Smoking status |  |  |  |  |
| Never | Ref. | Ref. | Ref. | Ref. |
| Former | -0.31 (-0.75, 0.13) | 0.173 | 0.61 (0.39, 0.84) | <0.001^***^ |
| Current | 1.16 (0.70, 1.63) | <0.001^***^ | 1.93 (1.67, 2.19) | <0.001^***^ |
| BMI | 0.39 (0.36, 0.43) | <0.001^***^ | 0.19 (0.17, 0.20) | <0.001^***^ |
| Total energy intake | 0.88 (0.70, 1.06) | <0.001^***^ | 0.43 (0.33, 0.53) | <0.001^***^ |
| CMDS |  |  |  |  |
| Stage 2 | Ref. | Ref. | Ref. | Ref. |
| Stage 3 | 0.64 (0.17, 1.11) | 0.009^**^ | 0.45 (0.2, 0.69) | <0.001^***^ |
| Stage 4 | 3.54 (3.08, 4.01) | <0.001^***^ | 3.07 (2.85, 3.28) | <0.001^***^ |
| CCI | 0.98 (0.83, 1.13) | <0.001^***^ | 0.76 (0.69, 0.82) | <0.001^***^ |
| CVD |  |  |  |  |
| No | Ref. | Ref. | Ref. | Ref. |
| Yes | 1.45 (0.87, 2.02) | <0.001^***^ | 2.05 (1.82, 2.29) | <0.001^***^ |
| Dysglycemia |  |  |  |  |
| Normal | Ref. | Ref. | Ref. | Ref. |
| IGT | -1.40 (-2.67, -0.12) | 0.035^*^ | -0.12 (-0.66, 0.43) | 0.678 |
| IFG | 0.37 (-0.35, 1.08) | 0.315 | 0.84 (0.45, 1.22) | <0.001^***^ |
| DM | 6.08 (5.51, 6.65) | <0.001^***^ | 4.14 (3.87, 4.41) | <0.001^***^ |
| Hyperlipidemia |  |  |  |  |
| No | Ref. | Ref. | Ref. | Ref. |
| Yes | 0.54 (0.10, 0.97) | 0.017^*^ | -0.15 (-0.35, 0.06) | 0.176 |
| CKD |  |  |  |  |
| No | Ref. | Ref. | Ref. | Ref. |
| Yes | 6.28 (5.83, 6.74) | <0.001^***^ | 3.44 (3.23, 3.65) | <0.001^***^ |
| Asthma |  |  |  |  |
| No | Ref. | Ref. | Ref. | Ref. |
| Yes | 0.84 (0.30, 1.37) | 0.003^**^ | 0.36 (0.06, 0.66) | 0.022* |
| MetS |  |  |  |  |
| No | Ref. | Ref. | Ref. | Ref. |
| Yes | 4.01 (3.69, 4.34) | <0.001^***^ | 2.11 (1.93, 2.30) | <0.001^***^ |
| Hypertension |  |  |  |  |
| No | Ref. | Ref. | Ref. | Ref. |
| Yes | 4.31 (3.97, 4.65) | <0.001^***^ | 2.04 (1.87, 2.21) | <0.001^***^ |
| Anti-hypertensive Drug |  |  |  |  |
| No | Ref. | Ref. | Ref. | Ref. |
| Yes | 1.90 (1.34, 2.47) | <0.001^***^ | 0.77 (0.50, 1.04) | <0.001^***^ |
| Anti-hyperlipidemic Drug |  |  |  |  |
| No | Ref. | Ref. | Ref. | Ref. |
| Yes | -0.82 (-1.33, -0.30) | 0.002^**^ | 1.25 (1.02, 1.48) | <0.001^***^ |

**Abbreviations:** CI = confidence interval; FPL = federal poverty level; IFG = Impaired Fasting Glycaemia; IGT = Impaired Glucose Tolerance; DM = diabetes mellitus; CCI = Charlson Comorbidity Index; CVD = cardiovascular diseases; DM = diabetes mellitus; MetS = metabolic syndrome; CKD = chronic kidney disease; KDMAccel = KDM BioAge Acceleration; PhenoAgeAccel = PhenoAge acceleration.

**Notes:** *P*-value: *, <0.05; **, <0.01; ***, <0.001. Cut-off values of DII tertiles were 0.9 and 2.6. The unit of total energy intake is “1000 kcal/day”.

**Supplementary Table 4. The effect of DII as continuous variable on aging acceleration.**

| **Models** | **β (95%CI)** | ***P*-value** |
| --- | --- | --- |
| **Crude Model** |  |  |
| KDMAccel | 0.33 (0.22, 0.44) | <0.001^***^ |
| PhenoAgeAccel | 0.24 (0.19, 0.29) | <0.001^***^ |
| **Model 1** |  |  |
| KDMAccel | 0.45 (0.34, 0.55) | <0.001^***^ |
| PhenoAgeAccel | 0.38 (0.33, 0.43) | <0.001^***^ |
| **Model 2** |  |  |
| KDMAccel | 0.51 (0.39, 0.63) | <0.001^***^ |
| PhenoAgeAccel | 0.30 (0.25, 0.35) | <0.001^***^ |
| **Model 3** |  |  |
| KDMAccel | 0.38 (0.29, 0.48) | <0.001^***^ |
| PhenoAgeAccel | 0.32 (0.27, 0.36) | <0.001^***^ |

**Abbreviations:** CI = confidence interval; KDMAccel = KDM BioAge Acceleration; PhenoAgeAccel = PhenoAge acceleration.

**Notes:** Model 1 adjusted for age, gendersex, ethnicity, and household income; model 2 adjusted for sex, BMI, and total energy intake; Model 3 adjusted age, sex, ethnicity, household income, smoking status, BMI, total energy intake, CCI, CVD, hypertension, dysglycemia, MetS, hyperlipidemia, asthma, CKD, anti-hypertensive drug use, and anti-hyperlipidemic drug use. *P*-value: *, <0.05; **, <0.01; ***, <0.001.

**Supplementary Table 5. Subgroup analyses for the effect of DII as continuous variable on aging acceleration.**

| **Subgroup** | **N** | **KDMAccel** | | | **PhenoAgeAccel** | | |
| --- | --- | --- | --- | --- | --- | --- | --- |
|  |  | **β (95%CI)** | ***P*-value** | ***P* for interaction** | **β (95%CI)** | ***P*-value** | ***P* for interaction** |
| Age |  |  |  | <0.001^***^ |  |  | 0.324 |
| <60 | 9874 | 0.32 (0.22, 0.43) | <0.001^***^ |  | 0.31 (0.25, 0.37) | <0.001^***^ |  |
| ≥60 | 6807 | 0.46 (0.28, 0.63) | <0.001^***^ |  | 0.31 (0.24, 0.38) | <0.001^***^ |  |
| Gender |  |  |  | 0.049^*^ |  |  | 0.043^*^ |
| Female | 8647 | 0.33 (0.19, 0.47) | <0.001^***^ |  | 0.28 (0.21, 0.35) | <0.001^***^ |  |
| Male | 8034 | 0.43 (0.32, 0.55) | <0.001^***^ |  | 0.35 (0.30, 0.40) | <0.001^***^ |  |
| Ethnicity |  |  |  | 0.037* |  |  | <0.001^***^ |
| White | 8678 | 0.39 (0.27, 0.51) | 0.003^**^ |  | 0.33 (0.28, 0.38) | <0.001^***^ |  |
| Black | 3095 | 0.37 (0.14, 0.60) | <0.001^***^ |  | 0.28 (0.14, 0.41) | <0.001^***^ |  |
| Mexican | 1537 | 0.20 (-0.10, 0.50) | 0.202 |  | 0.39 (0.25, 0.52) | <0.001^***^ |  |
| Others | 3371 | 0.54 (0.30, 0.78) | <0.001^***^ |  | 0.15 (0, 0.30) | 0.056 |  |
| Household income |  |  |  | 0.170 |  |  | 0.149 |
| ≤130% FPL | 4813 | 0.23 (0.05, 0.40) | 0.015^*^ |  | 0.30 (0.21, 0.39) | <0.001^***^ |  |
| 130%-350% FPL | 6585 | 0.42 (0.26, 0.59) | <0.001^***^ |  | 0.28 (0.20, 0.36) | <0.001^***^ |  |
| >350% FPL | 5283 | 0.41 (0.29, 0.54) | <0.001^***^ |  | 0.34 (0.27, 0.41) | <0.001^***^ |  |
| BMI |  |  |  | 0.323 |  |  | 0.904 |
| <30 kg/m^2^ | 9608 | 0.43 (0.31, 0.56) | <0.001^***^ |  | 0.34 (0.27, 0.41) | <0.001^***^ |  |
| ≥30 kg/m^2^ | 7073 | 0.32 (0.16, 0.48) | <0.001^***^ |  | 0.27 (0.20, 0.34) | <0.001^***^ |  |
| Smoking status |  |  |  | 0.454 |  |  | 0.919 |
| Never | 8442 | 0.40 (0.27, 0.52) | <0.001^***^ |  | 0.28 (0.23, 0.34) | <0.001^***^ |  |
| Former | 4734 | 0.28 (0.11, 0.46) | 0.002^**^ |  | 0.29 (0.20, 0.37) | <0.001^***^ |  |
| Current | 3505 | 0.47 (0.24, 0.70) | <0.001^***^ |  | 0.40 (0.27, 0.52) | <0.001^***^ |  |
| CCI |  |  |  | 0.180 |  |  | 0.550 |
| 0 | 8422 | 0.40 (0.28, 0.52) | <0.001^***^ |  | 0.33 (0.27, 0.38) | <0.001^***^ |  |
| >0 | 8259 | 0.35 (0.20, 0.49) | <0.001^***^ |  | 0.30 (0.24, 0.36) | <0.001^***^ |  |
| CMDS |  |  |  | 0.003^**^ |  |  | 0.100 |
| Stage 2 | 9966 | 0.39 (0.28, 0.51) | <0.001^***^ |  | 0.32 (0.26, 0.37) | <0.001^***^ |  |
| Stage 3 | 2164 | 0.42 (0.2, 0.63) | <0.001^***^ |  | 0.31 (0.21, 0.41) | <0.001^***^ |  |
| Stage 4 | 4551 | 0.34 (0.09, 0.59) | 0.009^**^ |  | 0.31 (0.21, 0.42) | <0.001^***^ |  |
| Dysglycemia |  |  |  | 0.095 |  |  | 0.037^*^ |
| Normal | 12404 | 0.41 (0.31, 0.51) | <0.001^***^ |  | 0.32 (0.27, 0.37) | <0.001^***^ |  |
| IFG | 719 | 0.46 (0.07, 0.85) | 0.023* |  | 0.40 (0.21, 0.59) | <0.001^***^ |  |
| IGT | 301 | 0.29 (-0.30, 0.87) | 0.326 |  | 0.04 (-0.26, 0.34) | 0.808 |  |
| DM | 3257 | 0.27 (-0.03, 0.56) | 0.079 |  | 0.29 (0.17, 0.42) | <0.001^***^ |  |
| Hyperlipidemia |  |  |  | 0.691 |  |  | 0.776 |
| No | 2850 | 0.37 (0.12, 0.63) | 0.005^**^ |  | 0.36 (0.25, 0.46) | <0.001^***^ |  |
| Yes | 13831 | 0.39 (0.28, 0.49) | <0.001^***^ |  | 0.31 (0.26, 0.36) | <0.001^***^ |  |
| MetS |  |  |  | 0.049* |  |  | 0.223 |
| No | 11075 | 0.41 (0.30, 0.52) | <0.001^***^ |  | 0.32 (0.27, 0.38) | <0.001^***^ |  |
| Yes | 5606 | 0.36 (0.16, 0.56) | <0.001^***^ |  | 0.31 (0.23, 0.40) | <0.001^***^ |  |
| Hypertension |  |  |  | <0.001^***^ |  |  | 0.036^*^ |
| No | 7945 | 0.34 (0.23, 0.45) | <0.001^***^ |  | 0.31 (0.24, 0.37) | <0.001^***^ |  |
| Yes | 8736 | 0.40 (0.24, 0.55) | <0.001^***^ |  | 0.32 (0.25, 0.38) | <0.001^***^ |  |
| CVD |  |  |  | <0.001^***^ |  |  | 0.007^**^ |
| No | 14479 | 0.37 (0.27, 0.47) | <0.001^***^ |  | 0.31 (0.26, 0.35) | <0.001^***^ |  |
| Yes | 2202 | 0.46 (0.18, 0.74) | 0.002^**^ |  | 0.38 (0.25, 0.51) | <0.001^***^ |  |

**Abbreviations:** CI = confidence interval; FPL = federal poverty level; IFG = Impaired Fasting Glycaemia; IGT = Impaired Glucose Tolerance; DM = diabetes mellitus; CCI = Charlson Comorbidity Index; CVD = cardiovascular diseases; DM = diabetes mellitus; MetS = metabolic syndrome; CKD = chronic kidney disease; KDMAccel = KDM BioAge Acceleration; PhenoAgeAccel = PhenoAge acceleration.

**Notes**: The effect size (β) was computed with linear regression. Adjusted for age, sex, ethnicity, household income, smoking status, BMI, total energy intake, CCI, CVD, hypertension, dysglycemia, MetS, hyperlipidemia, asthma, CKD, anti-hypertensive drug use, and anti-hyperlipidemic drug use. *P*-value: *, <0.05; **, <0.01; ***, <0.001.

**Supplementary Table 6. Subgroup analyses for the effect of DII as tertile variable on KDMAccel.**

| **Subgroup** | **N** | **First Tertile *vs* Second Tertile** | | **First Tertile *vs* Third Tertile** | | ***P* for interaction** |
| --- | --- | --- | --- | --- | --- | --- |
|  |  | **β (95%CI)** | ***P*-value** | **β (95%CI)** | ***P*-value** |  |
| Age |  |  |  |  |  | <0.001^***^ |
| <60 years | 9874 | 0.71 (0.27, 1.14) | <0.001^***^ | 1.23 (0.71, 1.75) | <0.001^***^ |  |
| ≥60 years | 6807 | 1.68 (1.01, 2.34) | <0.001^***^ | 1.77 (0.98, 2.56) | <0.001^***^ |  |
| Gender |  |  |  |  |  | 0.205 |
| Female | 8647 | 0.76 (0.21, 1.32) | 0.009^**^ | 1.28 (0.66, 1.89) | <0.001^***^ |  |
| Male | 8034 | 1.20 (0.71, 1.68) | <0.001^***^ | 1.57 (0.94, 2.2) | <0.001^***^ |  |
| Ethnicity |  |  |  |  |  | 0.042^*^ |
| White | 8678 | 1.22 (0.72, 1.72) | <0.001^***^ | 1.55 (0.98, 2.13) | 0.0012^**^ |  |
| Black | 3095 | 0.60 (-0.30, 1.50) | 0.199 | 1.60 (0.64, 2.57) | <0.001^***^ |  |
| Mexican | 3371 | 0.76 (0.05, 1.47) | 0.041* | 1.09 (0.15, 2.04) | 0.578 |  |
| Others | 1537 | 0.13 (-1.28, 1.55) | 0.854 | 0.48 (-0.99, 1.95) | 0.022^*^ |  |
| Household income |  |  |  |  |  | 0.331 |
| ≤130% FPL | 4813 | 0.40 (-0.28, 1.07) | 0.256 | 0.81 (0.16, 1.46) | 0.012^*^ |  |
| 130%-350% FPL | 6585 | 1.00 (0.42, 1.58) | 0.001** | 1.48 (0.69, 2.27) | <0.001^***^ |  |
| >3.5% FPL | 5283 | 1.23 (0.71, 1.75) | <0.001^***^ | 1.7 (1.04, 2.35) | <0.001^***^ |  |
| Smoking status |  |  |  |  |  | 0.303 |
| Never | 8442 | 0.98 (0.47, 1.50) | <0.001^***^ | 1.55 (0.97, 2.13) | <0.001^***^ |  |
| Former | 4734 | 1.16 (0.48, 1.83) | 0.001** | 0.92 (0.11, 1.73) | 0.002^**^ |  |
| Current | 3505 | 0.76 (-0.07, 1.58) | 0.077 | 1.7 (0.68, 2.73) | 0.025^*^ |  |
| BMI |  |  |  |  |  | 0.033^*^ |
| <30 kg/m^2^ | 9608 | 0.81 (0.36, 1.26) | <0.001^***^ | 1.55 (0.94, 2.17) | <0.001^***^ |  |
| ≥30 kg/m^2^ | 7073 | 1.33 (0.75, 1.91) | <0.001^***^ | 1.35 (0.68, 2.01) | <0.001^***^ |  |
| CCI |  |  |  |  |  | 0.052 |
| 0 | 8422 | 1.16 (0.71, 1.60) | <0.001^***^ | 1.45 (0.87, 2.03) | <0.001^***^ |  |
| > 0 | 8259 | 0.84 (0.31, 1.37) | 0.003^**^ | 1.39 (0.71, 2.06) | <0.001^***^ |  |
| Dysglycemia |  |  |  |  |  | 0.180 |
| Normal | 12404 | 0.97 (0.56, 1.37) | <0.001^***^ | 1.59 (1.10, 2.07) | <0.001^***^ |  |
| IFG | 719 | 0.47 (-0.91, 1.85) | 0.510 | 1.44 (-0.20, 3.08) | 0.072 |  |
| IGT | 301 | 1.84 (-1.26, 4.94) | 0.259 | 1.05 (-1.75, 3.84) | 0.516 |  |
| DM | 3257 | 1.62 (0.41, 2.82) | 0.011^*^ | 1.08 (-0.26, 2.41) | 0.130 |  |
| Hyperlipidemia |  |  |  |  |  | 0.197 |
| No | 2850 | 1.40 (0.52, 2.28) | 0.003^**^ | 1.10 (0.03, 2.17) | 0.045^*^ |  |
| Yes | 13831 | 0.94 (0.55, 1.34) | <0.001^***^ | 1.52 (1.02, 2.02) | <0.001^***^ |  |
| MetS |  |  |  |  |  | 0.116 |
| No | 11075 | 0.98 (0.54, 1.42) | <0.001^***^ | 1.55 (1.04, 2.05) | <0.001^***^ |  |
| Yes | 5606 | 1.22 (0.53, 1.91) | <0.001^***^ | 1.39 (0.49, 2.30) | 0.003^**^ |  |
| CMDS |  |  |  |  |  | 0.011^*^ |
| Stage 2 | 9966 | 0.89 (0.46, 1.32) | <0.001^***^ | 1.5 (0.99, 2.02) | <0.001^***^ |  |
| Stage 3 | 2164 | 1.18 (0.28, 2.08) | 0.013^*^ | 1.39 (0.36, 2.41) | 0.010^**^ |  |
| Stage 4 | 4551 | 1.54 (0.66, 2.41) | <0.001^***^ | 1.36 (0.25, 2.47) | 0.019^*^ |  |
| Hypertension |  |  |  |  |  | <0.001^***^ |
| No | 7945 | 0.69 (0.24, 1.14) | 0.004^**^ | 1.32 (0.81, 1.84) | <0.001^***^ |  |
| Yes | 8736 | 1.36 (0.78, 1.93) | <0.001^***^ | 1.51 (0.79, 2.24) | <0.001^***^ |  |
| CVD |  |  |  |  |  | <0.001^***^ |
| No | 14283 | 0.86 (0.47, 1.26) | <0.001^***^ | 1.42 (0.96, 1.88) | <0.001^***^ |  |
| Yes | 2398 | 2.46 (1.36, 3.56) | <0.001^***^ | 1.99 (0.72, 3.26) | 0.003^**^ |  |

**Abbreviations:** CI = confidence interval; FPL = federal poverty level; IFG = Impaired Fasting Glycaemia; IGT = Impaired Glucose Tolerance; DM = diabetes mellitus; CCI = Charlson Comorbidity Index; CVD = cardiovascular diseases; DM = diabetes mellitus; MetS = metabolic syndrome, CKD = chronic kidney disease; KDMAccel = KDM BioAge Acceleration; PhenoAgeAccel = PhenoAge acceleration.

**Notes**: The effect size (β) of DII on KDMAccel for each subgroup was computed with linear regression. The first tertile was considered as reference category. Adjusted for age, sex, ethnicity, household income, smoking status, BMI, total energy intake, CCI, CVD, hypertension, dysglycemia, MetS, hyperlipidemia, asthma, CKD, anti-hypertensive drug use, and anti-hyperlipidemic drug use. *P*-value: *, <0.05; **, <0.01; ***, <0.001.

**Supplementary Table 7. Subgroup analyses for the effect of DII (tertile) on PhenoAgeAccel.**

| **Subgroup** | **N** | **First Tertile *vs* Second Tertile** | | **First Tertile *vs* Third Tertile** | | ***P* for interaction** |
| --- | --- | --- | --- | --- | --- | --- |
|  |  | **β (95%CI)** | ***P*-value** | **β (95%CI)** | ***P*-value** |  |
| Age |  |  |  |  |  | 0.057 |
| <60 years | 9874 | 0.51 (0.28, 0.74) | <0.001^***^ | 1.17 (0.9, 1.45) | <0.001^***^ |  |
| ≥60 years | 6807 | 0.86 (0.6, 1.11) | <0.001^***^ | 1.21 (0.9, 1.51) | <0.001^***^ |  |
| Gender |  |  |  |  |  | 0.083 |
| Female | 8647 | 0.51 (0.25, 0.77) | <0.001^***^ | 1.07 (0.76, 1.37) | <0.001^***^ |  |
| Male | 8034 | 0.71 (0.47, 0.95) | <0.001^***^ | 1.36 (1.12, 1.61) | <0.001^***^ |  |
| Ethnicity |  |  |  |  |  | <0.001^***^ |
| White | 8678 | 0.74 (0.5, 0.98) | <0.001^***^ | 1.31 (1.05, 1.57) | <0.001^***^ |  |
| Black | 3095 | 0.28 (-0.17, 0.73) | 0.229 | 1.07 (0.53, 1.6) | <0.001^***^ |  |
| Mexican | 3371 | 0.88 (0.42, 1.33) | <0.001^***^ | 1.21 (0.69, 1.73) | <0.001^***^ |  |
| Others | 1537 | 0.01 (-0.68, 0.70) | 0.972 | 0.35 (-0.34, 1.03) | 0.323 |  |
| Household income |  |  |  |  |  | 0.037 |
| ≤130% FPL | 4813 | 0.25 (-0.14, 0.64) | 0.208 | 0.9 (0.5, 1.29) | <0.001^***^ |  |
| 130%-350% FPL | 6585 | 0.46 (0.19, 0.74) | 0.002^**^ | 1.06 (0.7, 1.41) | <0.001^***^ |  |
| >350% FPL | 5283 | 0.86 (0.56, 1.16) | <0.001^***^ | 1.43 (1.07, 1.8) | <0.001^***^ |  |
| Smoking status |  |  |  |  |  | 0.422 |
| Never | 8442 | 0.64 (0.37, 0.91) | <0.001^***^ | 1.14 (0.88, 1.41) | <0.001^***^ |  |
| Former | 4734 | 0.66 (0.35, 0.97) | <0.001^***^ | 1.12 (0.76, 1.47) | <0.001^***^ |  |
| Current | 3505 | 0.44 (0.05, 0.84) | 0.0304^*^ | 1.3 (0.79, 1.81) | <0.001^***^ |  |
| BMI |  |  |  |  |  | 0.067 |
| <30 kg/m^2^ | 9608 | 0.53 (0.3, 0.75) | <0.001^***^ | 1.27 (0.94, 1.59) | <0.001^***^ |  |
| ≥30 kg/m^2^ | 7073 | 0.76 (0.48, 1.05) | <0.001^***^ | 1.12 (0.84, 1.4) | <0.001^***^ |  |
| CCI |  |  |  |  |  | 0.141 |
| 0 | 8422 | 0.56 (0.31, 0.81) | <0.001^***^ | 1.26 (0.99, 1.53) | <0.001^***^ |  |
| >0 | 8259 | 0.74 (0.45, 1.02) | <0.001^***^ | 1.14 (0.85, 1.42) | <0.001^***^ |  |
| CMDS |  |  |  |  |  | 0.033* |
| Stage 2 | 9966 | 0.51 (0.29, 0.74) | <0.001^***^ | 1.23 (0.97, 1.49) | <0.001^***^ |  |
| Stage 3 | 2164 | 0.65 (0.26, 1.04) | <0.001^***^ | 1.08 (0.64, 1.52) | <0.001^***^ |  |
| Stage 4 | 4551 | 1.02 (0.59, 1.46) | <0.001^***^ | 1.19 (0.76, 1.63) | <0.001^***^ |  |
| Dysglycemia |  |  |  |  |  | 0.036^*^ |
| Normal | 12404 | 0.56 (0.36, 0.77) | <0.001^***^ | 1.25 (1.02, 1.48) | <0.001^***^ |  |
| IFG | 719 | 0.52 (-0.12, 1.15) | 0.09 | 1.45 (0.56, 2.34) | 0.00181** |  |
| IGT | 301 | 0.31 (-1.14, 1.76) | 0.678 | 0.09 (-1.32, 1.49) | 0.931 |  |
| DM | 3257 | 1.11 (0.55, 1.67) | <0.001^***^ | 1.12 (0.56, 1.68) | <0.001^***^ |  |
| Hyperlipidemia |  |  |  |  |  | 0.206 |
| No | 2850 | 0.99 (0.56, 1.42) | <0.001^***^ | 1.21 (0.72, 1.7) | <0.001^***^ |  |
| Yes | 13831 | 0.57 (0.35, 0.78) | <0.001^***^ | 1.21 (0.98, 1.45) | <0.001^***^ |  |
| MetS |  |  |  |  |  | 0.160 |
| No | 11075 | 0.55 (0.33, 0.78) | <0.001^***^ | 1.25 (0.98, 1.51) | <0.001^***^ |  |
| Yes | 5606 | 0.82 (0.51, 1.13) | <0.001^***^ | 1.19 (0.85, 1.53) | <0.001^***^ |  |
| Hypertension |  |  |  |  |  | 0.002^**^ |
| No | 7945 | 0.42 (0.14, 0.69) | 0.004^**^ | 1.17 (0.89, 1.45) | <0.001^***^ |  |
| Yes | 8736 | 0.86 (0.62, 1.11) | <0.001^***^ | 1.25 (0.95, 1.54) | <0.001^***^ |  |
| CVD |  |  |  |  |  | <0.001^***^ |
| No | 14283 | 0.52 (0.32, 0.72) | <0.001^***^ | 1.17 (0.95, 1.39) | <0.001^***^ |  |
| Yes | 2398 | 1.51 (0.98, 2.03) | <0.001^***^ | 1.61 (1.08, 2.14) | <0.001^***^ |  |

**Abbreviations:** CI = confidence interval; FPL = federal poverty level; IFG = Impaired Fasting Glycaemia; IGT = Impaired Glucose Tolerance; DM = diabetes mellitus; CCI = Charlson Comorbidity Index; CVD = cardiovascular diseases; DM = diabetes mellitus; MetS = metabolic syndrome, CKD = chronic kidney disease; KDMAccel = KDM BioAge Acceleration; PhenoAgeAccel = PhenoAge acceleration.

**Notes**: The effect size (β) of DII on PhenoAgeAccel for each subgroup was computed with linear regression. The first tertile was considered as reference category. Adjusted for age, sex, ethnicity, household income, smoking status, BMI, total energy intake, CCI, CVD, hypertension, dysglycemia, MetS, hyperlipidemia, asthma, CKD, anti-hypertensive drug use, and anti-hyperlipidemic drug use. *P*-value: *, <0.05; **, <0.01; ***, <0.001.

**Supplementary Table 8. Sensitivity analyses for the effect of DII on aging acceleration without complex survey design.**

| **Model** | **DII** | **KDMAccel** | | **PhenoAgeAccel** | |
| --- | --- | --- | --- | --- | --- |
|  |  | **β (95%CI)** | ***P*-value** | **β (95%CI)** | ***P*-value** |
| Crude Model | Continuous | 0.40 (0.31, 0.49) | <0.001^***^ | 0.24 (0.20, 0.29) | <0.001^***^ |
|  | Tertile |  |  |  |  |
|  | First Tertile | Ref. | Ref. | Ref. | Ref. |
|  | Second Tertile | 0.93 (0.54, 1.32) | <0.001^***^ | 0.44 (0.25, 0.63) | <0.001^***^ |
|  | Third Tertile | 1.50 (1.11, 1.89) | <0.001^***^ | 0.93 (0.74, 1.12) | <0.001^***^ |
|  | *P* for trend | <0.001^***^ |  | <0.001^***^ |  |
| Model 1 | Continuous | 0.46 (0.37, 0.55) | <0.001^***^ | 0.37 (0.32, 0.41) | <0.001^***^ |
|  | Tertile |  |  |  |  |
|  | First Tertile | Ref. | Ref. | Ref. | Ref. |
|  | Second Tertile | 1.07 (0.68, 1.45) | <0.001^***^ | 0.72 (0.54, 0.90) | <0.001^***^ |
|  | Third Tertile | 1.73 (1.34, 2.12) | <0.001^***^ | 1.40 (1.21, 1.58) | <0.001^***^ |
|  | *P* for trend | <0.001^***^ |  | <0.001^***^ |  |
| Model 2 | Continuous | 0.62 (0.52, 0.72) | <0.001^***^ | 0.40 (0.36, 0.45) | <0.001^***^ |
|  | Tertile |  |  |  |  |
|  | First Tertile | Ref. | Ref. | Ref. | Ref. |
|  | Second Tertile | 1.28 (0.89, 1.67) | <0.001^***^ | 0.73 (0.54, 0.91) | <0.001^***^ |
|  | Third Tertile | 2.31 (1.89, 2.73) | <0.001^***^ | 1.54 (1.34, 1.74) | <0.001^***^ |
|  | *P* for trend | <0.001^***^ |  | <0.001^***^ |  |
| Model 3 | Continuous | 0.40 (0.31, 0.49) | <0.001^***^ | 0.31 (0.27, 0.35) | <0.001^***^ |
|  | Tertile |  |  |  |  |
|  | First Tertile | Ref. | Ref. | Ref. | Ref. |
|  | Second Tertile | 0.88 (0.54, 1.23) | <0.001^***^ | 0.59 (0.43, 0.75) | <0.001^***^ |
|  | Third Tertile | 1.39 (1.01, 1.76) | <0.001^***^ | 1.13 (0.95, 1.31) | <0.001^***^ |
|  | *P* for trend | <0.001^***^ |  | <0.001^***^ |  |

**Notes:** Model 1 adjusted for age, gender, ethnicity, and household income; model 2 adjusted for gender, BMI, and total energy intake; Model 3 adjusted age, gender, ethnicity, household income, smoking status, BMI, total energy intake, CCI, CVD, hypertension, dysglycemia, MetS, hyperlipidemia, asthma, CKD, anti-hypertensive drug use, and anti-hyperlipidemic drug use.

**Supplementary Table 9. Sensitivity analyses for the effect of DII on aging acceleration using imputed data.**

| **Model** | **DII** | **KDMAccel** | | **PhenoAgeAccel** | |
| --- | --- | --- | --- | --- | --- |
|  |  | **β (95%CI)** | ***P*-value** | **β (95%CI)** | ***P*-value** |
| Crude Model | Continuous | 0.33 (0.22, 0.44) | <0.001^***^ | 0.24 (0.19, 0.29) | <0.001^***^ |
|  | Tertile |  |  |  |  |
|  | First Tertile | Ref. | Ref. | Ref. | Ref. |
|  | Second Tertile | 0.87 (0.41, 1.33) | <0.001^***^ | 0.46 (0.24, 0.67) | <0.001^***^ |
|  | Third Tertile | 1.26 (0.77, 1.75) | <0.001^***^ | 0.93 (0.70, 1.15) | <0.001^***^ |
|  | *P* for trend | <0.001^***^ |  | <0.001^***^ |  |
| Model 1 | Continuous | 0.45 (0.34, 0.55) | <0.001^***^ | 0.38 (0.33, 0.43) | <0.001^***^ |
|  | Tertile |  |  |  |  |
|  | First Tertile | Ref. | Ref. | Ref. | Ref. |
|  | Second Tertile | 1.12 (0.66, 1.58) | <0.001^***^ | 0.77 (0.57, 0.97) | <0.001^***^ |
|  | Third Tertile | 1.72 (1.22, 2.22) | <0.001^***^ | 1.50 (1.27, 1.73) | <0.001^***^ |
|  | *P* for trend | <0.001^***^ |  | <0.001^***^ |  |
| Model 2 | Continuous | 0.63 (0.51, 0.74) | <0.001^***^ | 0.42 (0.37, 0.47) | <0.001^***^ |
|  | Tertile |  |  |  |  |
|  | First Tertile | Ref. | Ref. | Ref. | Ref. |
|  | Second Tertile | 1.44 (0.99, 1.89) | <0.001^***^ | 0.80 (0.59, 1.01) | <0.001^***^ |
|  | Third Tertile | 2.42 (1.88, 2.96) | <0.001^***^ | 1.64 (1.40, 1.87) | <0.001^***^ |
|  | *P* for trend | <0.001^***^ |  | <0.001^***^ |  |
| Model 3 | Continuous | 0.38 (0.30, 0.47) | <0.001^***^ | 0.32 (0.27, 0.36) | <0.001^***^ |
|  | Tertile |  |  |  |  |
|  | First Tertile | Ref. | Ref. | Ref. | Ref. |
|  | Second Tertile | 0.90 (0.54, 1.25) | <0.001^***^ | 0.57 (0.39, 0.75) | <0.001^***^ |
|  | Third Tertile | 1.43 (1.03, 1.82) | <0.001^***^ | 1.19 (1.00, 1.38) | <0.001^***^ |
|  | *P* for trend | <0.001^***^ |  | <0.001^***^ |  |

**Notes:** Model 1 adjusted for age, gender, ethnicity, and household income; model 2 adjusted for gender, BMI, and total energy intake; Model 3 adjusted age, gender, ethnicity, household income, smoking status, BMI, total energy intake, CCI, CVD, hypertension, dysglycemia, MetS, hyperlipidemia, asthma, CKD, anti-hypertensive drug use, and anti-hyperlipidemic drug use.

**Supplementary Table 10. Sensitivity analyses for the effect of DII on aging acceleration as categorized variables.**

| **Models** | **DII** | **KDMAccel** | | **PhenoAgeAccel** | |
| --- | --- | --- | --- | --- | --- |
|  |  | **OR (95%CI)** | ***P*-value** | **OR (95%CI)** | ***P*-value** |
| Crude Model | Continuous | 1.06 (1.03, 1.09) | <0.001^***^ | 1.10 (1.08, 1.13) | <0.001^***^ |
|  | Tertile |  |  |  |  |
|  | First Tertile | Ref. | Ref. | Ref. | Ref. |
|  | Second Tertile | 1.21 (1.09, 1.34) | <0.001^***^ | 1.26 (1.15, 1.38) | <0.001^***^ |
|  | Third Tertile | 1.23 (1.09, 1.38) | <0.001^***^ | 1.43 (1.29, 1.60) | <0.001^***^ |
|  | *P* for trend | <0.001^***^ |  | <0.001^***^ |  |
| Model 1 | Continuous | 1.08 (1.05, 1.12) | <0.001^***^ | 1.16 (1.13, 1.19) | <0.001^***^ |
|  | Tertile |  |  |  |  |
|  | First Tertile | Ref. | Ref. | Ref. | Ref. |
|  | Second Tertile | 1.27 (1.14, 1.41) | <0.001^***^ | 1.40 (1.28, 1.54) | <0.001^***^ |
|  | Third Tertile | 1.34 (1.18, 1.52) | <0.001^***^ | 1.75 (1.56, 1.97) | <0.001^***^ |
|  | *P* for trend | <0.001^***^ |  | <0.001^***^ |  |
| Model 2 | Continuous | 1.10 (1.07, 1.14) | <0.001^***^ | 1.12 (1.09, 1.15) | <0.001^***^ |
|  | Tertile |  |  |  |  |
|  | First Tertile | Ref. | Ref. | Ref. | Ref. |
|  | Second Tertile | 1.30 (1.16, 1.45) | <0.001^***^ | 1.31 (1.19, 1.45) | <0.001^***^ |
|  | Third Tertile | 1.39 (1.22, 1.59) | <0.001^***^ | 1.52 (1.35, 1.72) | <0.001^***^ |
|  | *P* for trend | <0.001^***^ |  | <0.001^***^ |  |
| Model 3 | Continuous | 1.09 (1.05, 1.13) | <0.001^***^ | 1.16 (1.12, 1.19) | <0.001^***^ |
|  | Tertile |  |  |  |  |
|  | First Tertile | Ref. | Ref. | Ref. | Ref. |
|  | Second Tertile | 1.31 (1.16, 1.48) | <0.001^***^ | 1.41 (1.26, 1.57) | <0.001^***^ |
|  | Third Tertile | 1.35 (1.16, 1.57) | <0.001^***^ | 1.72 (1.49, 1.98) | <0.001^***^ |
|  | *P* for trend | <0.001^***^ |  | <0.001^***^ |  |

**Notes:** Model 1 adjusted for age, gender, ethnicity, and household income; model 2 adjusted for gender, BMI, and total energy intake; Model 3 adjusted age, gender, ethnicity, household income, smoking status, BMI, total energy intake, CCI, CVD, hypertension, dysglycemia, MetS, hyperlipidemia, asthma, CKD, anti-hypertensive drug use, and anti-hyperlipidemic drug use.

# Supplementary Methods

# 2.1 KDM Biological Age and KDMAccel

$$KDM Age=\frac{\sum_{i=1}^{n} \left( x_{i}-q_{i} \right)\frac{k_{i}}{s_{i}^{2}}+\frac{Chronological Age}{s_{BA}^{2}}}{\sum_{i=1}^{n} {(\frac{k_{i}}{s_{i}})}^{2}+\frac{1}{s_{BA}^{2}}}$$

$$KDMAccel=KDM Age-Chronological Age$$

Where *x* is the value of biomarker *i* measured for an individual. For each biomarker *i*, the parameters *k*, *q*, and *s* are estimated from a regression of chronological age on the biomarker in the reference sample. The *q*, *k*, and *s* are the regression intercept, slope, and root mean squared error, respectively. *s_BA_* is a scaling factor equal to the square root of the variance in chronological age explained by the biomarker set in the reference sample. The *q*, *k*, and *s* for each biomarker *i* were as follows:

| **Biomarkers** | **Female** | | | **Male** | | |
| --- | --- | --- | --- | --- | --- | --- |
|  | ***k*** | ***q*** | ***s*** | ***k*** | ***q*** | ***s*** |
| Albumin, g/L | -0.002187197 | 4.157074783 | 0.34276922 | -0.00725206 | 4.577019066 | 0.343255342 |
| Alkaline phosphatase, u/L | 0.629927313 | 54.95837587 | 27.88506641 | 0.222890703 | 76.03706101 | 25.49458647 |
| CRP (ln), mg/dl | 0.0011776 | 0.303289327 | 0.279985824 | 0.002781679 | 0.154975132 | 0.21909775 |
| Total cholesterol, mg/dl | 1.314791516 | 146.3495243 | 41.23107254 | 0.384942117 | 190.3257889 | 40.85523984 |
| Creatinine (ln), mg/dl | 0.001734239 | 0.466950058 | 0.088193423 | 0.001580101 | 0.587415694 | 0.09772561 |
| HbA1c, % | 0.022953617 | 4.449792928 | 1.01186265 | 0.017329423 | 4.731511106 | 0.921120203 |
| Systolic blood pressure, mmHg | 0.796155042 | 85.51138091 | 16.84732312 | 0.557382604 | 101.089652 | 15.8792247 |
| Blood urea nitrogen, mg/dl | 0.14484357 | 6.193569619 | 4.235136641 | 0.10113296 | 10.10763561 | 4.827920343 |
| Uric acid, mg/dl | 0.025099998 | 3.534643882 | 1.29292154 | 0.002664833 | 5.942965218 | 1.372528458 |
| Lymphocyte percent | -0.016095247 | 34.73356778 | 8.567578358 | -0.093601372 | 37.55095697 | 8.604743322 |
| Mean cell volume, fl | 0.051379381 | 86.27481765 | 5.789227001 | 0.047325418 | 87.72206357 | 5.222452794 |
| WBC count, 1000 cells/ul | -0.007737116 | 7.543679511 | 2.134273367 | 0.001751592 | 7.059668657 | 2.112217805 |

# 2.2 PhenoAge Biological Age and PhenoAgeAccel

$$Phenotypic Age=141.8254+\frac{\ln\left[ -0.005807831\times\ln\left( 1-M \right) \right]}{0.08742185}$$

$$PhenoAgeAccel=Phenotypic Age-Chronological Age$$

Where:

$$M=1-exp(\frac{-1.386933\times exp(xb)}{0.007250078})$$

And:

$$xb=-18.264084479-0.037550125\times albumin\_gL+0.001977458\times Alkaline Phosphatase+ 0.178246261\times ln(CRP)-0.001046473\times Total cholesterol+0.846406370\times ln (Creatinine) +0.161423930\times HbA1c+0.007376810\times Systolic blood pressure-0.013646067\times Blood urea nitrogen+0.049875933\times Uric acid-0.010733596\times Lymphocyte Percent+0.021307220\times Mean Cell Volume+0.062521994\times White Blood Cell Count+0.078026776\times Chronological Age$$

# 2.3 Dietary inflammatory index

$$DII=\sum_{i=1}^{n} {FS}_{i}$$

Where:

$$FS=(2\times CDF\left( \frac{x-s_{1}}{SD} \right)-1)\times s_{2}$$

The *x* is the value of food parameter *i* measured for an individual. The *s_1_* was the value of “global daily mean intake”, *s_2_* was the value of “overall food parameter-specific inflammatory effect score”, and *FS* was the value of “food parameter-specific DII score”. CDF was cumulative distribution function. The DII was the “dietary inflammatory index”. The s1, s2, and SD for each food parameter were as follows:

| Food parameter | Overall food parameter-specific inflammatory effect score | Global daily mean intake (units/day) | SD |
| --- | --- | --- | --- |
| Alcohol, g/day | -0.278 | 13.98 | 3.72 |
| β-carotene, μg/day | -0.584 | 3718 | 1720 |
| Caffeine, g/day | -0.11 | 8.05 | 6.67 |
| Carbohydrates, g/day | 0.097 | 272.2 | 40 |
| Cholesterol, mg/day | 0.11 | 279.4 | 51.2 |
| Total energy intake, kcal/day | 0.18 | 2056 | 338 |
| Dietary fibre, g/day | -0.663 | 18.8 | 4.9 |
| Folic acid, μg/day | -0.19 | 273 | 70.7 |
| Fe, mg/day | 0.032 | 13.35 | 3.71 |
| Mg, mg/day | -0.484 | 310.1 | 139.4 |
| MUFA, g/day | -0.009 | 27 | 6.1 |
| Niacin, mg/day | -0.246 | 25.9 | 11.77 |
| n-3 fatty acids, g/day | -0.436 | 1.06 | 1.06 |
| n-6 fatty acids, g/day | -0.159 | 10.8 | 7.5 |
| Protein, g/day | 0.021 | 79.4 | 13.9 |
| PUFA, g/day | -0.337 | 13.88 | 3.76 |
| Riboflavin, mg/day | -0.068 | 1.7 | 0.79 |
| Saturated fat, g/day | 0.373 | 28.6 | 8 |
| Selenium, μg/day | -0.191 | 67 | 25.1 |
| Total fat, g/day | 0.298 | 71.4 | 19.4 |
| Thiamin, mg/day | -0.098 | 1.7 | 0.66 |
| Vitamin B12, μg/day | 0.106 | 5.15 | 2.7 |
| Vitamin B6, mg/day | -0.365 | 1.47 | 0.74 |
| Vitamin A, RE/day | -0.401 | 983.9 | 518.6 |
| Vitamin C, mg/day | -0.424 | 118.2 | 43.46 |
| Vitamin D, μg/day | -0.446 | 6.26 | 2.21 |
| Vitamin E, mg/day | -0.419 | 8.73 | 1.49 |
| Zinc, mg/day | -0.313 | 9.84 | 2.19 |

# 2.4 Charlson comorbidity index (CCI)

The weights of each comorbidity for CCI were as follows.

| Disease | Weight |
| --- | --- |
| Diabetes mellitus | 1 |
| Diabetic retinopathy | 2 |
| Kidney failure | 2 |
| Kidney stones | 2 |
| Heart failure | 1 |
| Stroke | 1 |
| Chronic obstructive pulmonary disease | 1 |
| Asthma | 1 |
| Chronic bronchitis | 1 |
| Liver disease | 2 |
| Rheumatoid arthritis | 1 |
| Bladder cancer | 2 |
| Bone cancer | 2 |
| Brain cancer | 2 |
| Breast cancer | 2 |
| Cervical cancer | 2 |
| Colon cancer | 2 |
| Esophageal cancer | 2 |
| Gallbladder carcinoma | 2 |
| Kidney cancer | 2 |
| Leukemia | 2 |
| Liver cancer | 2 |
| Lung cancer | 2 |
| Lymphomas | 2 |
| Melanoma | 2 |
| Nervous system cancer | 2 |
| Oral cancer | 2 |
| Ovarian cancer | 2 |
| Pancreatic cancer | 2 |
| Prostatic cancer | 2 |
| Rectal cancer | 2 |
| Skin cancer (non-melanoma) | 2 |
| Other skin cancer | 2 |
| Soft tissue cancer | 2 |
| Stomach cancer | 2 |
| Testicular cancer | 2 |
| Thyroid cancer | 2 |
| Tracheal carcinoma | 2 |
| Endometrial cancer | 2 |
| Other cancer | 2 |
